# Bulk and Single-Cell Profiling of Breast Tumors Identifies TREM-1 as a Dominant Immune Suppressive Marker Associated With Poor Outcomes

**DOI:** 10.3389/fonc.2021.734959

**Published:** 2021-12-08

**Authors:** Ashok K. Pullikuth, Eric D. Routh, Kip D. Zimmerman, Julia Chifman, Jeff W. Chou, Michael H. Soike, Guangxu Jin, Jing Su, Qianqian Song, Michael A. Black, Cristin Print, Davide Bedognetti, Marissa Howard-McNatt, Stacey S. O’Neill, Alexandra Thomas, Carl D. Langefeld, Alexander B. Sigalov, Yong Lu, Lance D. Miller

**Affiliations:** ^1^ Department of Cancer Biology, Wake Forest School of Medicine, Winston Salem, NC, United States; ^2^ Lineberger Comprehensive Cancer Center, University of North Carolina at Chapel Hill, Chapel Hill, NC, United States; ^3^ Center for Precision Medicine, Wake Forest School of Medicine, Winston Salem, NC, United States; ^4^ Department of Mathematics and Statistics, American University, Washington, DC, United States; ^5^ Department of Biostatistics and Data Science, Wake Forest School of Medicine, Winston Salem, NC, United States; ^6^ The Comprehensive Cancer Center of Wake Forest University, Winston Salem, NC, United States; ^7^ Department of Radiation Oncology, University of Alabama-Birmingham, Birmingham, AL, United States; ^8^ Department of Biostatistics and Health Data Science, Indiana University School of Medicine, Indianapolis, IN, United States; ^9^ Center for Cancer Genomics and Precision Oncology, Wake Forest School of Medicine, Winston Salem, NC, United States; ^10^ Department of Biochemistry, Otago School of Medical Sciences, University of Otago, Dunedin, New Zealand; ^11^ Department of Molecular Medicine and Pathology and Maurice Wilkins Institute, Faculty of Medical and Health Sciences, The University of Auckland, Auckland, New Zealand; ^12^ Cancer Program, Sidra Medicine, Doha, Qatar & Department of Internal Medicine and Medical Specialties, University of Genoa, Genoa, Italy; ^13^ Surgical Oncology Service, Department of Surgery, Wake Forest School of Medicine, Winston Salem, NC, United States; ^14^ Department of Pathology, Wake Forest School of Medicine, Winston Salem, NC, United States; ^15^ Section of Hematology and Oncology, Department of Internal Medicine, Wake Forest Baptist Medical Center, Winston Salem, NC, United States; ^16^ SignaBlok, Inc. Shrewsbury, MA, United States; ^17^ Department of Microbiology & Immunology, Wake Forest School of Medicine, Winston Salem, NC, United States

**Keywords:** TREM-1, tumor infiltrating myeloid cells, transcriptomics, immune signature, cytokines, breast cancer, immune suppression

## Abstract

**Background:**

Triggering receptor expressed on myeloid cells (TREM)-1 is a key mediator of innate immunity previously associated with the severity of inflammatory disorders, and more recently, the inferior survival of lung and liver cancer patients. Here, we investigated the prognostic impact and immunological correlates of *TREM1* expression in breast tumors.

**Methods:**

Breast tumor microarray and RNAseq expression profiles (n=4,364 tumors) were analyzed for associations between gene expression, tumor immune subtypes, distant metastasis-free survival (DMFS) and clinical response to neoadjuvant chemotherapy (NAC). Single-cell (sc)RNAseq was performed using the 10X Genomics platform. Statistical associations were assessed by logistic regression, Cox regression, Kaplan-Meier analysis, Spearman correlation, Student’s t-test and Chi-square test.

**Results:**

In pre-treatment biopsies, *TREM1* and known TREM-1 inducible cytokines (IL1B, IL8) were discovered by a statistical ranking procedure as top genes for which high expression was associated with reduced response to NAC, but only in the context of immunologically “hot” tumors otherwise associated with a high NAC response rate. In surgical specimens, *TREM1* expression varied among tumor molecular subtypes, with highest expression in the more aggressive subtypes (Basal-like, HER2-E). High *TREM1* significantly and reproducibly associated with inferior distant metastasis-free survival (DMFS), independent of conventional prognostic markers. Notably, the association between high *TREM1* and inferior DMFS was most prominent in the subset of immunogenic tumors that exhibited the immunologically hot phenotype and otherwise associated with superior DMFS. Further observations from bulk and single-cell RNAseq analyses indicated that *TREM1* expression was significantly enriched in polymorphonuclear myeloid-derived suppressor cells (PMN-MDSCs) and M2-like macrophages, and correlated with downstream transcriptional targets of TREM-1 (*IL8*, *IL-1B*, *IL6*, *MCP-*1, *SPP1, IL1RN, INHBA*) which have been previously associated with pro-tumorigenic and immunosuppressive functions.

**Conclusions:**

Together, these findings indicate that increased *TREM1* expression is prognostic of inferior breast cancer outcomes and may contribute to myeloid-mediated breast cancer progression and immune suppression.

## Introduction

The clinical behavior of cancer can be influenced by the composition and functional orientation of pro- and anti-tumor immune mediators within the tumor microenvironment (TME) (1, 2). In the TME and nearby lymphoid structures, tumor-reactive immune cells that favor tumor destruction (ie, cytotoxic T cells (CTLs), Natural Killer (NK) cells, T-helper (Th) and dendritic cells (DC)) can act to slow cancer growth and spread, and contribute directly to favorable therapeutic responses. However, this protective immunity is frequently counter-balanced by suppressive immune cell populations, such as myeloid-derived suppressor cells (MDSCs), regulatory T-cells (T_REGs_) and tumor associated macrophages (TAMs) that favor immunosuppression and immune escape (2, 3). Furthermore, paracrine signaling cascades initiated by cytokines and growth factors produced by the latter cell types can also directly promote cancer cell growth, survival, and metastasis, thereby contributing directly to tumor progression (4).

Genome-wide gene expression profiling studies in solid tumors have provided context for understanding how complex multicellular immune interactions impact tumor aggressiveness (5–7). Recent reports by us and others have described immune gene signatures in breast tumors that comprise genes with specialized roles in immuno-biology, and that quantify the relative abundance and functional properties of distinct tumor-infiltrating leukocyte populations (8–12). Significantly, these immune gene signatures have been shown to correlate with patient survival outcomes (8, 13, 14), chemotherapy responsiveness (13, 15), and more recently, response to immunotherapies (16–18). Prognostic and therapy-predictive signatures identified to date constitute a diverse genetic fingerprint of innate and adaptive immunity, inclusive of B cells, T cells, DCs, CTLs, NK cells, macrophages, neutrophils, and mast cells (5, 6, 10, 19). Yet, what these signatures reveal about specific immune-regulatory mechanisms operative in the TME remains largely unknown.

In previous reports, we demonstrated that the significant associations between breast tumor immune subclasses (defined by effector immune gene signatures) and patient clinical outcomes was strongly dependent on cancer-intrinsic properties such as tumor proliferation rate and mutational burden (8, 14, 20), thereby implicating these phenotypes in immune-mediated breast cancer outcomes. These observations prompted us to use logistic regression to identify specific genes that may antagonize otherwise favorable immune-mediated outcomes, as such genes could hold promise as novel immunotherapeutic targets. Herein, we describe the discovery and characterization of one such candidate, the gene encoding Triggering Receptor Expressed on Myeloid Cells (TREM)-1, which emerged as a robust therapy-predictive and prognostic marker by unsupervised analyses in large expression profiling data sets. *TREM1* encodes a type I transmembrane receptor of the Ig superfamily expressed by effectors of innate immunity including neutrophils, monocytes and macrophages. The TREM-1 receptor is known to augment inflammatory signaling in response to infectious pathogens by promoting release of cytokines that modulate the activation, recruitment and survival of myeloid and lymphoid cells (21, 22), yet its function in cancer remains unclear.

In this report, we provide the first evidence that *TREM1* expression in breast tumors has negative prognostic and therapy-predictive implications for breast cancer patients, and that *TREM1* expression is mediated predominantly by breast tumor-infiltrating myeloid cells that may constitute a cell population that antagonizes anti-tumor immunity. Our findings raise the possibility that the targeting of TREM-1 signaling in breast tumors may represent a viable immunotherapeutic strategy.

## Materials and Methods

### Gene Expression Datasets

Gene expression profiles of breast tumors and monocytes were obtained from MIAME-compliant studies (23) that followed Institutional Review Board (IRB)-approved protocols. The assembly, normalization and clinical annotation of the MDACC-701 gene expression dataset has been described previously (15). Briefly, this data set consists of Affymetrix U133A microarray gene expression profiles of 701 breast tumor biopsies (acquired prior to administration of neoadjuvant chemotherapy) and corresponding clinical drug response annotation. Compilation of normalized gene expression data and clinical annotations constituting the breast tumor meta-cohort #1 (MC1) data set has been described previously (8, 20). Briefly, the MC1 data set consists of 1,954 patient samples and corresponding clinical annotations. Expression data were generated using the U133 series GeneChip microarray platform (Affymetrix) containing 22,268 probe sets common to U133A, U133A2 and U133 PLUS 2.0 array platforms. MC1 represents independent cohorts from 16 medical centers in the United States, Europe and Asia. The meta-cohort #2 (MC2) data set includes 616 breast tumor expression profiles from patients in the United States, Asia, Europe and New Zealand. For both cohorts, raw data (CEL files) were downloaded from the NCBI’s Gene Expression Omnibus (GEO) data repository and processed as previously described (8, 20). MC1 and MC2 were analyzed on the same Affymetrix platform, and RMA-normalized and batch corrected using R software package from the Bioconductor project (8, 20). Molecular subtype calls were assigned to MC1 and MC2 cohorts according to Nagalla et al. (8). To identify gene expression changes downstream of TREM-1 activation, the Dower et al. gene expression dataset (24) comparing expression profiles of human peripheral blood monocytes with TREM-1 activation (induced by ligation with agonist antibody, n=11 patients) to those of matched control-treated monocytes (isotype antibody, n=11 patients) was analyzed. Affymetrix U133 PLUS 2.0 CEL files were downloaded from the GEO data repository (accession GSE9988) and RMA-normalized as described above. Differential expression analyses were conducted using Wilcoxon rank-sum test. Genes with Benjamini-Hochberg FDR-adjusted p-values < 0.01 were considered significant.

### Tumor Immune Subclasses

*FID*, *WID* and *PID* (Favorable, Weak and Poor Immune Dispositions, respectively) are gene expression-based, tumor immune subclasses that reflect the continuum from immunologically “inflamed” (FID) to immune “cold” (PID) tumors (8, 20). The immune subclasses are based on the integration of scores for three distinct effector immune gene signatures, identified in a previous breast tumor study using the MC1 data set (8). These immune signatures reflect the relative abundance of tumor-infiltrating effector cells associated with cytolytic activity (*T/NK signature*; CD8+ T cells/NK cells), antigen presentation (*M/D signature*; myeloid/dendritic cells), and humoral immunity (*B/P signature*; B cells/plasma cells). Each immune signature is scored in tumors using the geometric mean of expression of a set of immune-specialized genes, and each signature was found to provide additive prognostic information in multivariable Cox regression models (8). Patients were assigned to immune subclasses as follows. For each of the three immune signatures, tumors were ranked into population tertiles according to their immune signature scores. Tumors with upper-tertile expression scores for all three signatures, simultaneously, are classified as FID; tumors with lowest-tertile expression scores for any one signature are classified as PID; and tumors with combined intermediate- and upper-tertile signature scores are classified as WID, as established previously (8, 20). Accordingly, patients with FID tumors were observed to experience significantly better distant metastasis-free survival than those with WID or PID tumors, while those with PID tumors experienced significantly worse survival than those with WID or FID tumors. In a similar vein, FID tumors (at biopsy) were found to exhibit a significantly higher rate of responsiveness to neoadjuvant chemotherapy as compared to WID or PID tumors, consistent with reports that breast tumors with elevated TIL at baseline show greater responsiveness to neoadjuvant chemotherapy (25, 26); while PID tumors showed a significantly poorer response to neoadjuvant treatment as compared to WID or FID tumors (15).

### Analysis of Gene Interactions With Immune Subclasses and Chemotherapy Response

Logistic regression was used to identify genes with antagonistic interactions with the effect of FID, WID and PID immune subclasses on tumor response to neoadjuvant chemotherapy. The MDACC-701 data set was utilized, and the analysis was restricted to the most well detected genes defined as being the probe sets whose mean expression levels across tumors were greater than the median of the mean expression among genes (n=11,108 probe sets). Tumor response was defined as favorable (Pathologic Complete Response (pCR) or Residual Cancer Burden (RCB) score = 0 or 1) or unfavorable (RCB score = 2 or 3) as previously described for this data set (15). In the logistic regression model, covariates included tumor response (0, 1), immune subclass (FID, WID, PID), patient age (continuous) and expression profile of each microarray gene (MG, dichotomous (using median as cut point)), while the interaction between MG expression and immune subclass was defined as being the parameter of interest. The magnitude of an interaction was represented by the regression coefficient of the product term. The logistic regression was run in R, and a Wald test was used to evaluate the statistical significance of each coefficient in the model. The Wald test computes a Z statistic as the ratio of the square of the regression coefficient to the square of the standard error of the coefficient and then computes significance. The unadjusted significance of discovered genes ranged from *P* = 0.05 to *P* = 0.00005, and gene ontology enrichment analysis was used to identify significantly-associated biological processes in the top genes ranked by Z statistic (Z values ≤ -2.0).

### Gene Ontology (GO) Term Enrichment

The DAVID Functional Annotation Tool (DAVID Bioinformatics Resources version 6.7, NIAID/NIH) (27) was used to study associations between gene sets and GO terms related to biological processes and pathways.

### Survival Analyses

Molecular and clinical markers were analyzed as continuous or categorical variables for significant associations with DMFS by Cox proportional hazards regression (including multivariable models containing clinical and molecular covariates) and Kaplan-Meier analysis. GraphPad Prism, Sigmaplot 12.0 and the R package “survival” (v3.3-2) were used for these analyses, with DMFS endpoints defined as “no evidence of distant recurrence” (censored) or “clinical evidence of distant recurrence” (event), within 10 years of diagnosis.

### Expression of *TREM1*-Associated Genes in Tissues and Various Cell Types

To compute tissue-specific gene enrichment scores, we used the procedure and data set described in Benita et al., (28) and the *limma* package of Bioconductor (29). Briefly, one compares each group to all others and computes the linear model coefficient for each pair, which is a measure of the difference between two groups. For each linear model coefficient, *limma* also computes an associated *P* value (Bonferroni-corrected *P* ≤ 0.05). Genes that are specific to one tissue subset will result in higher enrichment scores. Proportions of immune cell types in tumors of the MC1 data set were estimated from RNA transcripts according to the CIBERSORT method and the LM-22 gene matrix (547 genes that distinguish 22 human hematopoietic cell types) (19, 30). CIBERSORT employs a novel support vector regression (SVR) to deconvolve proportions of distinct cell types found within complex mixtures. The resultant cell proportion estimates were mean-centered and averaged within quartiles of *TREM1* expression. The R package *gplots* was used to visualize the cell proportion averages by heat map (31, 32). Cell proportion estimates were analyzed by Spearman’s rank-ordered correlation for significant associations with *TREM1* expression. *TREM1* expression in The Cancer Genome Atlas (TCGA)-breast cancer (BRCA) database was analyzed by TIMER (cistrome.org/TIMER/) (33) for significant correlations between *TREM1*, downstream target genes and markers of myeloid cell populations. The whole dataset (Breast Invasive Carcinoma; n = 1,093) and the Basal-like tumors (BRCA-Basal; n = 139) were utilized.

### Histological Analysis of TREM-1 and CD68 in Breast Tumor Specimens

Human breast tumor tissue microarrays consisting of a variety of invasive ductal and lobular carcinomas were fabricated in-house by the Wake Forest Baptist Comprehensive Cancer Center’s Tumor Tissue and Pathology Shared Resource and used in immunofluorescence staining studies as follows. Formalin-fixed paraffin-embedded (FFPE) sections (5 μm) on slides were deparaffinized, antigen retrieved with sodium citrate method, blocked with 2.5% heat-inactivated horse serum, and incubated with polyclonal goat anti-TREM-1 antibody (ab) (1:50; R&D systems #AF1278) and mouse anti-CD68 (pan-myeloid) monoclonal ab (1:100; DAKO #2015-08, Clone KP1) in a humid chamber overnight at 4°C. After washing with PBS-T (PBS with 0.05% Tween-20), sections were incubated with rabbit anti-goat-Alexa 546 (1:200; Invitrogen) and goat anti-mouse Alexa 488 (1:250; Invitrogen) for 2 hours at room temperature in a humid chamber. After rinsing with PBS-T, sections were stained with DAPI or DRAQ5 (1:1000, 5μM, Cell Signaling) for 30 min, rinsed and mounted in Prolong anti-fade (Invitrogen) or FluoroShield (Sigma). Negative control and isotype matched mouse IgG1 (DAKO # 2015-07) were used to confirm staining specificity. Slides were imaged on an Olympus VS-110 Virtual Microscopy System at the Wake Forest School of Medicine Virtual Microscopy Core Facility.

### Single-Cell RNAseq Analysis

Remnant surgical tissue from an AJCC stage III triple negative invasive ductal carcinoma resected at Wake Forest Baptist Medical Center (WFBMC) in Winston Salem, NC, was accessed *via* Wake Forest University Health Sciences Institutional Review Board protocol IRB00048977. Fresh tumor tissue was minced by razor blade into ~1 mm pieces and digested for 1 hr at 37°C with Human Dissociation Kit (Miltenyi Biotec, Auburn, CA) as per manufacturer’s instruction with the aid of gentleMACS Octo Dissociator fitted with heating collar (Miltenyi Biotec). Digested tissue was passed through a 70 µm strainer, washed and resuspended in RPMI plus 10% fetal bovine serum (FBS), then underlaid with Histopaque-1077 (Sigma-Aldrich) and centrifuged at 400 x g for 30 min. The resulting buffy coat was harvested and washed in RPMI plus 10% FBS, resuspended in the same media, and counted. Approximately 1 million cells were frozen in 90% FBS/10% DMSO. A single-cell cDNA library was generated from thawed cells exhibiting >80% viability using the 10X Genomics Chromium Controller (34) maintained by the Wake Forest Baptist Comprehensive Cancer Center’s Cancer Genomics Shared Resource. Indexed libraries were paired-end sequenced using an Illumina NextSeq 500 at an average read depth of 173,842 mapped reads per cell (n = 770 cells). Raw bcl and fastq data were demultiplexed, mapped to the GRCh38 human reference genome, and post-processed using Cell Ranger (10X Genomics) mkfastq pipelines and QC algorithms. Processed data files have been deposited in the Gene Expression Omnibus (GEO; www.ncbi.nlm. nih.gov/geo/) and can be accessed through GEO Series accession number GSE188600. Cells that expressed less than 400 genes or that contained mitochondrial reads comprising >20% of their total count of unique molecular identifiers were excluded. T-distributed stochastic neighbor embedding (t-SNE) (35) was used to reduce data dimensionality and cluster cells based on gene expression patterns. In accordance with the Cell Ranger R software, the statistical significance of genes differentially expressed between cell clusters or identified cell populations was assessed by negative binomial exact test [for genes with low counts, based on sSeq method (36)] and fast asymptotic beta test [for genes with high counts, edgeR method (37)]. Genes with Benjamini-Hochberg FDR-adjusted p-values < 0.01 were considered significant. A linear normalized expression value threshold > 0 was used to identify cells positive for *TREM1* expression or expression of other marker genes studied.

In parallel, we analyzed a publicly available single cell RNAseq dataset (the Bassez et al. dataset) that profiled 175,942 cells from 31 treatment-naïve primary breast tumors (European Genome-phenome Archive (EGA) accession no. EGAS00001004809). Consistent with the analysis of the TNBC specimen from WFBMC, this study also utilized the 10X Genomics Chromium system for single cell library production, followed by Illumina sequencing (NextSeq 500 and NovaSeq 6000 platforms), and utilized Cell Ranger data processing workflows and similar quality control filters for cell selection (38). Analysis of the Bassez et al. dataset was facilitated by the BioTuring BBrowser and associated analytical tools (39) and the Seurat v3 R toolkit (40).

## Results

### *TREM1* Expression Antagonizes Favorable Immune-Associated Chemotherapy Responses

To identify genes with potential to antagonize anti-tumor immunity, we investigated breast tumor *immune subclasses* predictive of neoadjuvant chemotherapy response. Previous studies have shown that the abundance of tumor infiltrating lymphocytes in breast tumors is predictive of chemotherapy response in the neoadjuvant setting (41, 42). In earlier studies we identified three breast tumor immune gene signatures that reflect the relative abundance of tumor-infiltrating effector cell populations associated with cytolytic activity (T/NK signature), antigen presentation (M/D signature) and humoral immunity (B/P signature), each with independent prognostic power (8). These signatures allowed discernment of three tumor immune subclasses, termed FID, WID and PID (i.e., Favorable, Weak and Poor Immune Dispositions, respectively), that reflect the continuum from immunologically “inflamed” (FID) to immune “cold” (PID) tumors (see *Materials and Methods*). These subclasses were reported previously to differ significantly in terms of patient survival (8, 14, 20) and response to neoadjuvant chemotherapy (15) with FID tumors associating with more favorable clinical outcomes as compared to PID tumors, consistent with protective anti-tumor immunity being associated with FID status. To discover genes expressed in the TME that antagonize immune-mediated protective effects, we utilized a retrospective microarray dataset, MDACC-701 (15), consisting of 701 breast tumor expression profiles from core or needle biopsies taken from patients prior to neoadjuvant chemotherapy. In this data set, the FID immune subclass was found to identify patients with a significantly higher chemotherapy response rate as compared to WID or PID tumors. Using logistic regression (see *Materials and Methods*), we ranked genes from the MDACC-701 dataset based on the magnitude of antagonistic interaction, where higher gene expression in the FID immune subclass tumors was associated with a decrease in favorable chemotherapy response. Ranking genes by Z statistic, we identified 195 genes (207 probe sets) with interaction Z values ≤ -2.0 (see **Supplemental Table 1**), and analyzed these genes for significant enrichment of Gene Ontology (GO) terms. The one significant biological process identified was Neutrophil Chemotaxis (univariate *P* = 5.8x10^-5^, Benjamini-Hochberg adjusted *P* = 0.06) which was supported by 7 genes. Among these genes, the most significant by Z statistic was *TREM1* [ranked #6 overall for negative Z value (Z = -3.3, *P* = 0.001, Wald test)]. Moreover, included among the seven genes were the two well-characterized TREM-1 target genes, *IL1B* and *CXCL8* (IL8), both of which showed a highly significant positive correlation with *TREM1* expression in the MDACC-701 dataset (Pearson and Spearman correlations >0.6; see **Supplemental Figure 1**).

**Figure 1:**
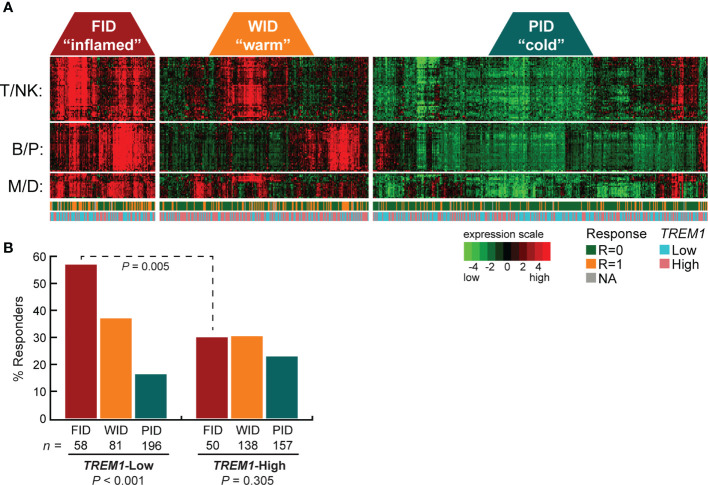
High *TREM1* expression in breast tumor biopsies associates with reduced efficacy of neoadjuvant chemotherapy in tumors with high effector immune infiltrates. The breast tumor biopsy expression profiling data set of Alistar et al. (15) was used to evaluate interactions between *TREM1* expression and neoadjuvant chemotherapy response within immune subclasses. **(A)** Heatmap of the genes comprising the T/NK (T cell/NK cell), B/P (B-cell/Plasma cell), and M/D (Myeloid/Dendritic cell) gene signatures are shown for tumors classified into the FID, WID and PID immune subclasses (see *Materials and Methods* for additional details). Tumor response to neoadjuvant chemotherapy (1 = responder, 0 = nonresponder) and tumor *TREM1* expression level (Low = below median, High = above median) are indicated beneath the heatmap. **(B)** The fraction of chemotherapy-responsive tumors (Y-axis) is shown for each immune subclass as a function of low or high *TREM1* (X-axis). Chemotherapy response rates were compared across immune subclasses (within low or high *TREM1* groups) and within immune subclasses (across low and high *TREM1* groups) by Chi-square test with Yates correction. Only the FID subclass exhibited a difference in chemotherapy response across *TREM1* low and high tumors (*P* = 0.005).

TREM-1 is a type-1 cell surface receptor expressed on cells of myeloid origin that triggers the transcriptional activation of numerous cytokines and chemokines in response to inflammatory signals, such as pathogen- and damage-associated molecular patterns (43). Moreover, TREM-1 was recently shown to inhibit anti-tumor immunity in a model of hepatocellular carcinoma (44). To further examine the relationship between *TREM1* expression and the therapy-predictive power of the immune subclasses, we stratified patients first according to subclass (FID, WID or PID), and then according to low or high *TREM1* expression (**Figure 1A**). We observed that the higher frequency of favorable response in FID tumors was dependent on low *TREM1* expression. Specifically, the frequency of favorable response was 1.9-fold higher in *TREM1*-low FID tumors as compared to *TREM1*-high FID tumors (*P* = 0.005, Chi-square test), while no difference in response was observed among WID or PID tumors in the context of *TREM1* low or high status (**Figure 1B** **)**.

### *High TREM1* Expression Predicts Inferior Distant Metastasis-Free Survival (DMFS)

**Figure 2:**
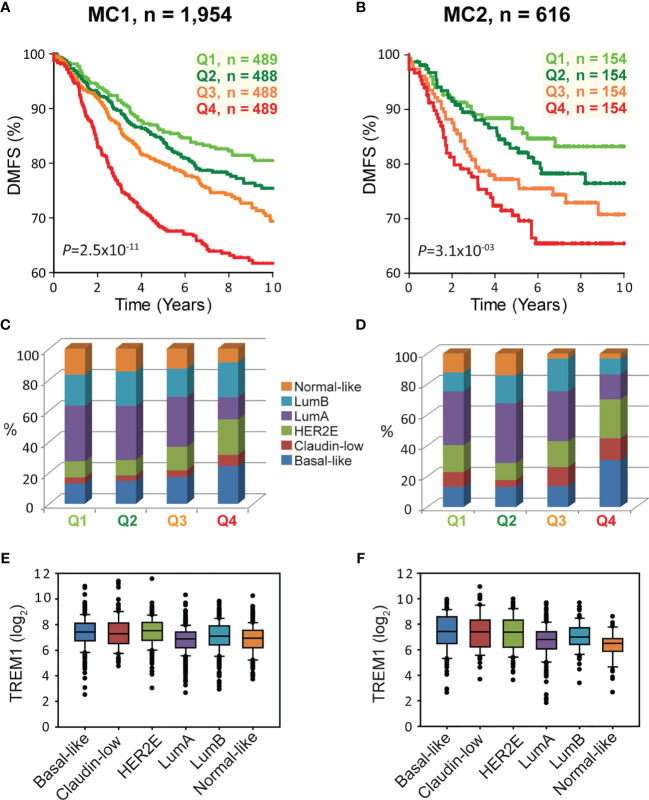
High *TREM1* is associated with greater risk of distant metastasis in two independent meta-cohorts. **(A, B)** Kaplan-Meier survival analysis of patients according to *TREM1* expression quartiles in **(A)** the MC1 cohort and **(B)** the MC2 cohort. Proportions of molecular subtypes within *TREM1* quartiles are shown for **(C)** the MC1 cohort and **(D)** the MC2 cohort. Box plots of *TREM1* expression distributions within molecular subtypes are shown for **(E)** the MC1 cohort and **(F)** the MC2 cohort.

To examine the association between *TREM1* expression and patient prognosis, we utilized a multi-institutional data set (Meta-Cohort 1 (MC1)) consisting of 1,954 breast tumor expression profiles characterized on the Affymetrix U133 platform and annotated for distant metastasis-free survival, molecular subtype, and other clinical and demographic features (8, 45). First, we subdivided the MC1 cohort into two evaluation cohorts (termed 977A and 977B, respectively) by randomizing the tumor expression profiles equally into two cohorts. Cox regression analysis was performed for each gene and within each evaluation cohort to identify genes with significant associations with DMFS. Genes significantly associated with DMFS (*P* < 0.1, Benjamini-Hochberg) in 977A (n = 3,094) and 977B (n = 3,304) were then used as input for hierarchical clustering of samples and genes (**Supplemental Figures 2A, B** **)**. Previously reported prognostic gene clusters were identified, including a cluster of inferior outcome-associated, proliferation-related genes that mirror cancer proliferative capacity (8) and several correlated clusters of immune-specialized genes that reflect infiltrating B cells, T cells and dendritic cells and associate with good survival outcomes (8). *TREM1* was found to be significantly inversely associated with DMFS in both 977A and 977B, and reproducibly clustered together with a set of co-expressed inferior outcome-associated genes enriched for myeloid cell functions including *SPP1*, *IL8* (*CXCL8*), *INHBA*, *GREM1*, *PLAU*, *PLAUR*, *AQP9*, *ADM*, *HPSE*, and *CTSL1*. Among the genes of this cluster, *TREM1* was found to have the most statistically significant association with inferior DMFS in both the 977A and 977B data sets (*P* = 5.3x10^-6^ and *P* = 1.6x10^-4^, respectively) (**Supplemental Figure 2C** **)**. To further study this association, patients from the full MC1 cohort were stratified into quartiles based on *TREM1* expression levels, and the quartiles were examined by Kaplan-Meier analysis. Patients whose tumors ranked in the highest quartile of *TREM1* (Q4) experienced poorer DMFS as compared to those ranked in lower quartiles (*P* = 2.5x10^-11^, log-rank test), with progressively worse DMFS observed for each increment of *TREM1* quartile (**Figure 2A**). This observation was reproducible in an independent breast cancer data set, Meta-Cohort 2 (MC2), consisting of 616 patients whose tumors were also profiled on the Affymetrix U133 platform (8, 20) (*P* = 3.1x10^-03^; **Figure 2B** **)**. Analysis of molecular subtypes showed that the highest quartile of *TREM1* expression (Q4) contained proportionally more Basal-like, Claudin-low and HER2E tumors, and fewer Luminal A (LumA) and Normal-like tumors, as compared to the lowest quartile (Q1) (**Figures 2C, D** **)**. However, *TREM1* expression distributions only showed moderate variation between different molecular subtypes, with decreased expression of *TREM1* observed in LumA and Normal-like tumors, in particular (**Figures 2E, F** **)**. Notably, we observed that *TREM1* expression associated with poorer DMFS within certain clinical and molecular subtypes. By univariate Cox regression analysis (**Table 1** **)**, *TREM1* was associated with DMFS in both estrogen receptor positive (ER+) and negative (ER-) breast cancers, as well as lymph node positive (LN+) and negative (LN-) disease. Within molecular subtypes, *TREM1* levels showed significant associations with DMFS in Basal-like, Luminal B (LumB) and Luminal A (LumA) tumor subtypes. We further examined the prognostic relevance of *TREM1* by adjusting for conventional variables in multivariable Cox models (**Table 2**). As a continuous variable, *TREM1* expression was associated with poorer DMFS, univariately (*P* = 1.9x10^-13^), as well as in the multivariable model (*P* = 3.2x10^-05^), where it remained significant in the presence of molecular subtype, immune subclass, histologic grade, tumor size, lymph node status, ER status, patient age, and treatment status.

**Table 1:** Cox proportional hazards regression analysis of *TREM1* in clinical and molecular subtypes of the MC1 cohort.

Characteristics	n^2^	Hazard Ratio (95% CI)^3^	P-value^4^
All	1954	1.40 (1.25-1.48)	1.9 X10^-13^
**ER/LN Status** ^1^			
ER^+^	1343	1.30 (1.17-1.44)	9.6 X10^-07^
ER^-^	401	1.44 (1.24-1.66)	1.4 X10^-06^
LN^+^	437	1.30 (1.12-1.50)	6.0 X10^-04^
LN^-^	1498	1.34 (1.24-1.51)	3.2 X10^-10^
**Molecular Subtype**			
Basal-like	334	1.55 (1.29-1.85)	1.9 X10^-06^
Luminal A	565	1.25 (1.00-1.55)	4.7 X10^-02^
Luminal B	399	1.19 (1.03-1.38)	2.2 X10^-02^
HER2E	281	1.18 (0.97-1.42)	9.3 X10^-01^
Claudin-low	92	1.13 (0.84-1.52)	4.3 X10^-01^
Normal-like	257	1.16 (0.84-1.60)	3.7 X10^-01^

^1^Estrogen receptor/lymph node status; ^2^number of patients; ^3^95% confidence interval; ^4^likelihood ratio test P-value.

**Table 2:** Cox proportional hazards regression analysis for associations with distant metastasis-free survival.

Variables	Univariable		Multivariable	
	Hazard Ratio (95% CI)^1^	*p-*value^2^	Hazard Ratio (95% CI)	*p-*value
*TREM1* (continuous)	1.36 (1.25-1.48)	1.9 X10^-13^	1.24 (1.12-1.37)	3.2 X10^-05^
PAM50: NL *vs*. Basal	2.74 (1.82-4.14)	1.5 X10^-06^	1.75 (1.00-3.07)	5.2 X10^-02^
NL *vs*. Claudin Low	2.75 (1.63-4.65)	1.6 X10^-04^	1.04 (0.51-2.17)	9.0 X10^-01^
NL *vs*. HER2E	3.60 (2.40-4.43)	8.7 X10^-10^	2.17 (1.27-3.71)	4.7 X10^-03^
NL *vs*. LumA	1.37 (0.90-2.06)	1.4 X10^-01^	0.96 (0.57-1.62)	8.8 X10^-01^
NL *vs*. LumB	3.56 (2.40-5.30)	2.7 X10^-10^	2.18 (1.31-3.64)	2.9 X10^-03^
Grade: I *vs*. II	2.50 (1.62-3.85)	3.1 X10^-05^	1.95 (1.22-3.13)	5.4 X10^-03^
I *vs*. III	4.30 (2.81-6.55) 4.287 (2.805,6.551)	1.7 X10^-11^	2.44 (1.51-3.96)	3.0 X10^-04^
T size: <20mm *vs*. 20-50mm	1.43 (1.18-1.73)	2.3 X10^-04^	1.40 (1.10-1.78)	6.8 X10^-03^
<20mm *vs*. >50mm	3.21 (2.11-4.90)	6.0 X10^-08^	2.89 (1.59-5.25)	5.0 X10^-04^
LN Status (-,+)	1.63 (1.34-1.98)	1.1 X10^-06^	1.88 (1.35-2.61)	2.0 X10^-04^
Age (≤40 yrs, >40 yrs)	0.66 (0.50-0.84)	2.6 X10^-03^	0.77 (0.57-1.05)	1.0 X10^-01^
ER Status (-,+)	0.68 (0.56-0.84)	3.6 X10^-04^	0.93 (0.68-1.26)	6.2 X10^-01^
Adjuvant Treatment (no, yes)	0.89 (0.74-1.06)	1.9 X10^-01^	0.55 (0.40-0.75)	2.3 X10^-04^
IMM: PID *vs*. WID	0.62 (0.50-0.77)	8.8 X10^-06^	0.62 (0.47-0.80)	3.8 X10^-04^
PID *vs*. FID	0.54 (0.50-0.77)	6.9 X10^-06^	0.47 (0.33-0.66)	1.2 X10^-05^

^1^95% confidence interval; ^2^likelihood ratio test p-value.

### *TREM1* Expression Antagonizes Immune-Dependent DMFS

**Figure 3:**
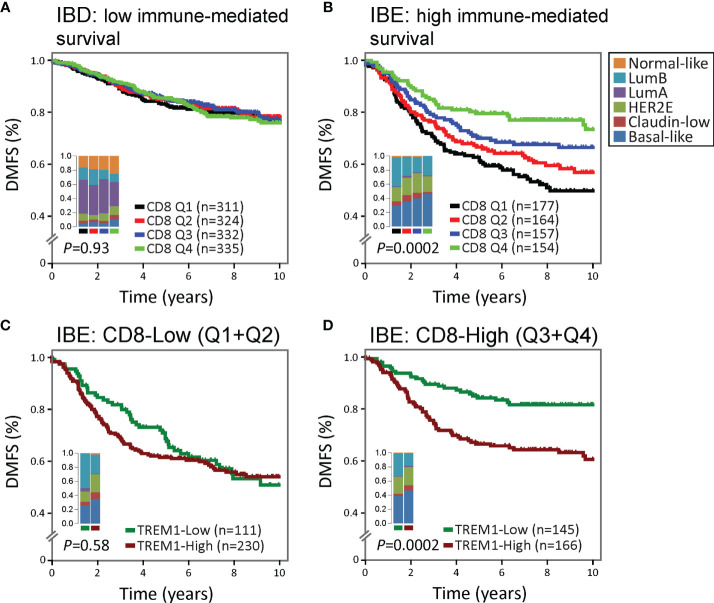
The association between high *TREM1* and increased metastatic risk is most significant in immunogenic tumors with DMFS-protective high CD8+ T cell infiltrates. The prognostic relevance of *TREM1* expression was examined in **(A)** poorly immunogenic tumors of the MC1 cohort with low proliferative capacity [classified previously as immune benefit-disabled (IBD) (20)], and **(B)** moderate to highly immunogenic tumors of the MC1 cohort with high proliferative capacity [classified previously as immune benefit-enabled (IBE) (20)]. Patients were stratified into DMFS survival curves based on the relative magnitude of tumor-infiltrating CD8+ T cells (CD8) estimated by the CIBERSORT algorithm (19). Q1, lowest CD8 quartile (black); Q2 and Q3, intermediate CD8 quartiles (red and blue); Q4, highest CD8 quartile (green). **(C, D)** Survival rates of patients with low (below-median) versus high (above-median) *TREM1* expression were compared in the context of low versus high CD8+ T cell fraction estimates. The most significant difference was observed in IBE tumors. *TREM1* expression level was not significantly associated with DMFS in IBE CD8-low tumors **(C)**, but showed marked significance in IBE CD8-high tumors **(D)**, where high *TREM1* expression was significantly associated with increased risk of distant metastasis.

We hypothesized that the negative association between *TREM1* expression and DMFS may reflect, in part, TREM-1-related immunoregulatory signaling that antagonizes favorable immune-mediated outcomes. In previous work, we showed that the FID, WID and PID immune subclasses exhibited a reproducible significant prognostic stratification in breast tumors classified as *immune benefit-enabled* (IBE), consisting mostly of highly proliferative Basal-like, HER2-enriched and Luminal B subtypes, but not in *immune benefit-disabled* (IBD) tumors defined by a reduced proliferative phenotype (20). Using the CIBERSORT algorithm (19) which deconvolves gene expression profiles from complex cellular mixtures and estimates the relative proportions of 22 different leukocyte types, we investigated the prognostic significance of *TREM1* in the context of the estimated proportions of CD8+ T cells in IBD and IBE breast tumors (**Figure 3**). CD8+ T cell scores were used to stratify the MC1 cohort into CD8+ T cell quartiles (Q1=lowest quartile, Q4=highest quartile). While higher quartiles of CD8+ T cells associated with improved DMFS in IBE tumors (*P* = 0.0002) (**Figures 3A, B** **)**, the prognostic power of *TREM1* was most significant in IBE CD8+ T cell-High tumors (defined as quartiles 3 and 4 combined; *P* = 0.0002) as compared to CD8+ T cell-Low tumors (defined as quartiles 1 and 2 combined; *P* = 0.58) (**Figures 3C, D** **)**, with TREM1-Low tumors exhibiting a more favorable DMFS. This observation is consistent with a possible role for TREM-1 in attenuating protective T cell-mediated immunity in breast cancer.

### *TREM1* Expression in the TME Is Associated With a Myeloid Phenotype

**Figure 4:**
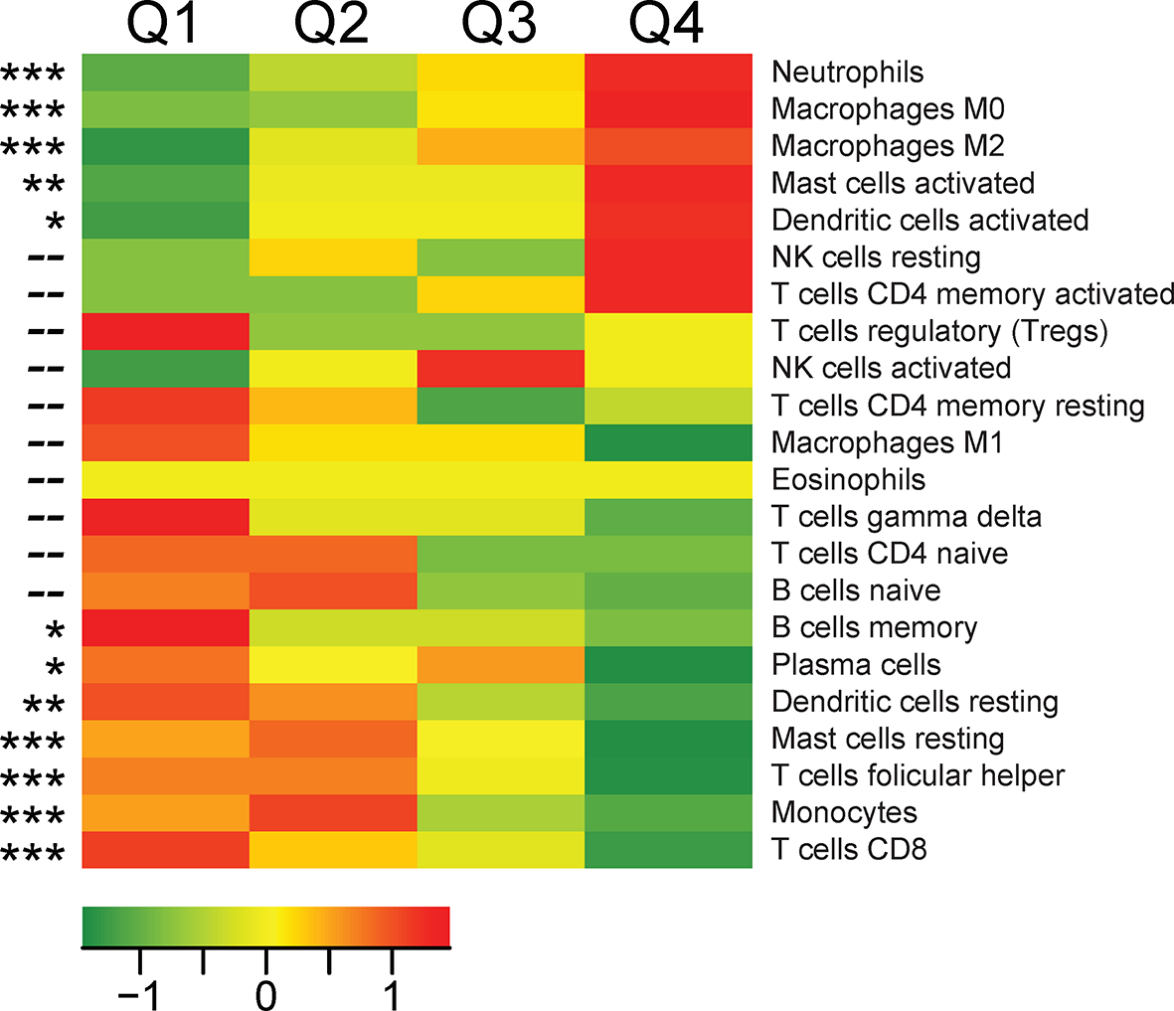
Associations between *TREM1* expression quartiles and abundance of leukocyte populations in breast tumors. For each tumor of the MC1 cohort, a CIBERSORT estimate of relative immune cell proportion was computed for each of 22 immune cell types. Heat map values represent the mean CIBERSORT immune cell proportions for each quartile of *TREM1* expression (mean-centered). Red color, high average cell proportion; green color, low average cell proportion. For cell types where cell abundance correlates positively or negatively with *TREM1* expression quartiles, Spearman correlation p-values are shown. *P < 0.05; **P < 0.01; ***P < 0.001.

To determine the cellular source of *TREM1* expression, we first analyzed the expression patterns of *TREM1* and the genes comprising the *TREM1*-associated gene cluster observed in breast tumors using the Gene Enrichment Profiler tool (28), which measures tissue-specific gene enrichment in 126 normal human cell subsets and tissues. This analysis showed that in non-malignant tissues, *TREM1* is highly enriched in macrophages and neutrophils (**Supplemental Figure 3**), consistent with previous reports (46, 47). Similarly, a number of genes comprising the *TREM1*-associated gene cluster that we identified by hierarchical clustering (ie, SPP1, PLAUR, AQP9, IL8 (CXCL8), ADM, UPP1, and INHBA) were also highly enriched in myeloid cells, and in lipopolysaccharide (LPS)-stimulated macrophages, in particular (**Supplemental Figure 3**), suggesting a common myeloid origin for *TREM1* and this gene expression cassette. In parallel, we investigated TREM-1 expression directly by immunofluorescence staining using a breast tumor tissue microarray consisting of >40 invasive ductal and lobular carcinomas positive or negative for the clinical markers ER, PGR and HER-2. Across tumor types, the predominant TREM-1 staining pattern observed was moderate to strong TREM-1 staining in a fraction of tumor-infiltrating myeloid cells that co-expressed CD68, with negative staining in tumor cells (pattern P-1, **Figure 6A**). In some instances we observed only CD68-positive cells negative for TREM-1 staining, which coincided with malignant (and normal) epithelium also negative for TREM-1 (P2). In more rare instances we observed TREM-1 staining in non-myeloid cells of tumors (P3), such as moderate TREM-1 staining in cancer cells (n=2), or moderate to high TREM1 staining in cells morphologically consistent with cancer-associated fibroblasts (n=3). To further investigate the relationship between *TREM1* and tumor infiltrating immune cells, we used the CIBERSORT algorithm to study the relationship between immune cell abundance estimates and *TREM1* expression quartiles in the MC1 cohort. As shown in **Figure 4**, a number of immune cell types showed marked proportional differences across *TREM1* expression quartiles. In the highest *TREM1* quartile (Q4), we observed significant enrichment of neutrophils, macrophages (M0 and M2, but not M1), mast cells (activated, but not resting) and dendritic cells (activated). By contrast, significant depletion in *TREM1* Q4 was observed for CD8+ T cells, monocytes, follicular helper T cells, mast cells (resting), dendritic cells (resting) and plasma and memory B cells. These observations are consistent with a positive correlation between high *TREM1* expression, increasing proportions of immunosuppressive myeloid cell types (M2 macrophages, neutrophils and mast cells) and decreasing proportions of anti-tumor effector cells (CD8+ T cells and follicular helper T cells).

**Figure 5:**
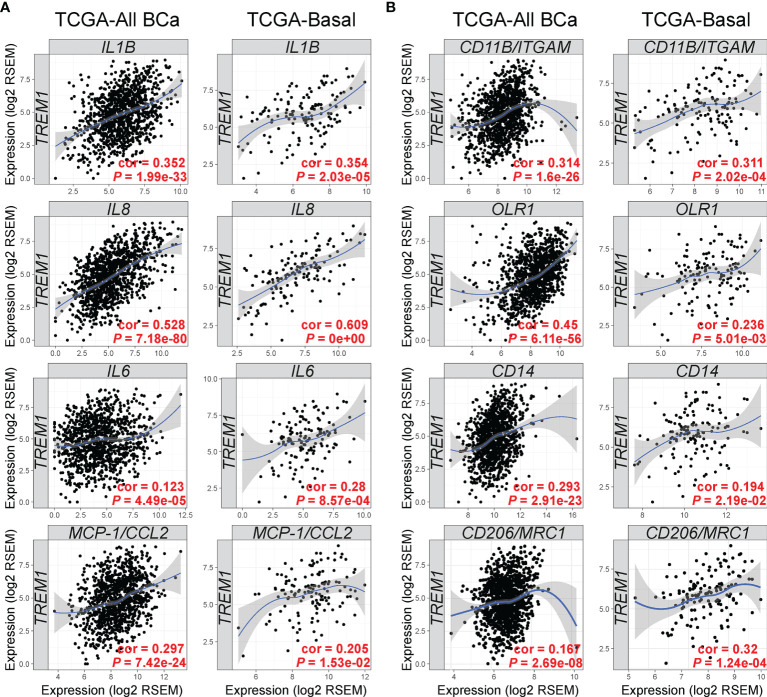
*TREM1* correlates with expression of TREM-1-inducible cytokines and markers of tumor-associated myeloid cell populations. The Tumor Immune Estimation Resource (TIMER) (33) was used to analyze gene expression correlations between *TREM1* and **(A)** its cognate cytokines and **(B)** markers of MDSCs and M2 macrophages. Correlations were analyzed in tumor populations representing all TCGA breast cancer (BCa) cases (n = 1,093) or the Basal-like/TNBC cases (n = 139) only. Spearman’s correlation coefficient (rho) and *P*-value are shown.

Next, we examined the TCGA breast cancer (BRCA) RNAseq data set for *TREM1* correlations with known target genes (**Figure 5A**) and myeloid markers (**Figure 5B**). Side-by-side analyses were conducted for the set of all breast tumors, and for the basal-like breast tumors, alone, since they showed the highest average expression of TREM-1 as compared to other subtypes in the MC1 and MC2 cohorts. Significant positive correlations were identified between intratumoral *TREM1* expression and downstream TREM-1 target genes that encode cytokines with direct roles in the recruitment and activation of pro-tumorigenic myeloid cells (*IL1B*, *IL8*, *IL6*, *MCP-1/CCL2*), as well as markers of pro-tumorigenic myeloid cells including *CD11B/ITGAM* (a general MDSC marker), *CD14* [marker for monocytic (M)-MDSCs (48)], *OLR1* [marker for PMN-MDSC (49)] and *CD206/MRC1* (M2 macrophage marker).

### Single-Cell RNA Sequencing Verifies TREM-1 Signaling and Association With MDSC and TAM Compartments in Breast Tumors

**Figure 6:**
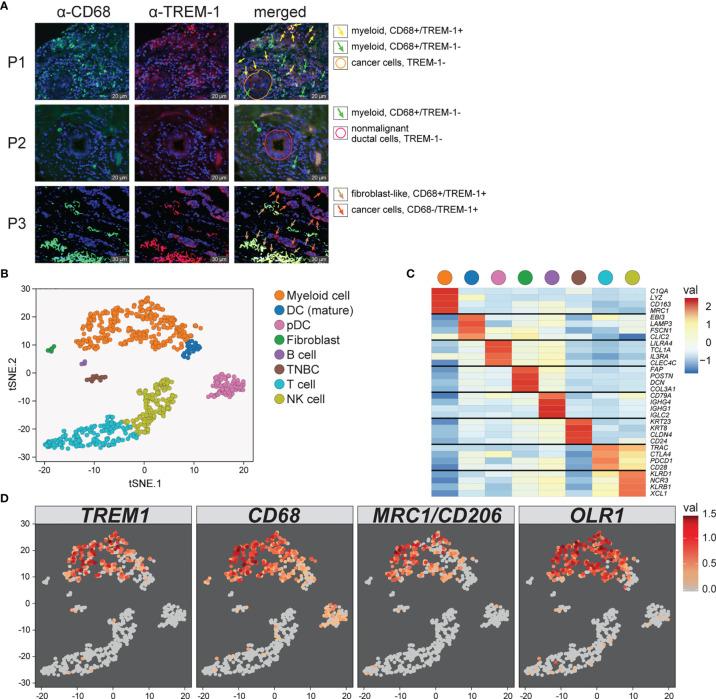
Characterization of *TREM1* expression in the breast tumor microenvironment by IF and single-cell RNAseq. **(A)** Representative patterns (P) of TREM-1 (red) and CD68 (green) immunofluorescent staining in breast tumor sections. DRAQ5-stained nuclei are shown in blue pseudocolor. **(B)** Single-cell RNA sequencing was performed on freshly dissociated cells of a stage III primary triple negative breast tumor. Expression profiles of 770 cells were K-means clustered and resolved spatially in a tSNE plot. **(C)** Cell identities in **(B)** were assigned based on significant expression of canonical marker genes. Mean-centered averages of cluster-specific gene expression are shown in the heatmap. **(D)** Relative expression levels of *TREM1*, *CD68*, *MRC1* (M2 macrophage marker) and *OLR1* (MDSC marker) are shown.

**Figure 7:**
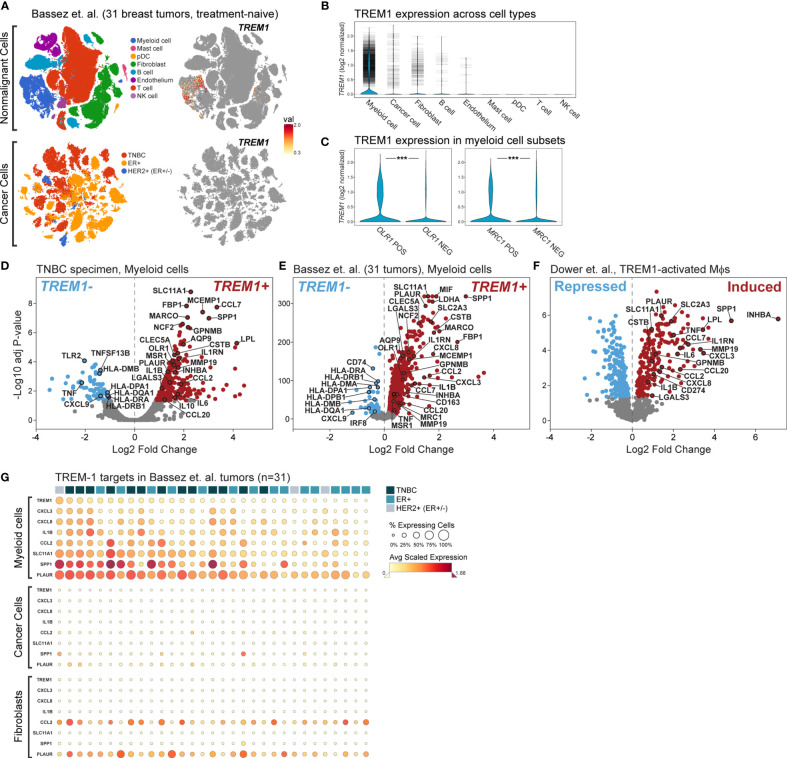
Single cell analysis of *TREM1* and correlated genes in a breast tumor panel. **(A)** Shown are tSNE plots derived from the Bassez et al. single cell RNAseq data for 175,942 cells from 31 primary breast tumors comprising TNBC (n = 13), ER^+^ (n = 15) and HER2^+^/ER^+/-^ (n = 3) subtypes. Plots are shown for nonmalignant cells (upper panels) and cancer cells (lower panels). The magnitude of *TREM1* expression across cell populations is illustrated spatially (right panels), and in **(B)** violin plots across cell types. Horizontal tick marks denote individual cell measurements. **(C)** *TREM1* expression is compared between myeloid cells grouped according to positive or negative expression of OLR1 (MDSC marker, left panel) or MRC1 (M2 TAM marker, right panel). ***P < 0.001. Volcano plots of genes differentially expressed between myeloid cells positive or negative for *TREM1*expression are shown for **(D)** the WFBMC TNBC specimen [204 genes differentially expressed between *TREM1+* cells (n=123) and *TREM1–* cells (n = 178)], and **(E)** the set of 31 breast tumors of Bassez et al. [3,223 genes differentially expressed between *TREM1+* cells (n = 3,333) and *TREM1–* cells (n = 13,152)]. **(F)** Volcano plot of genes differentially expressed between TREM-1-activated (agonist Ab-treated) and control (isotype Ab-treated) human blood monocytes of Dower et al. [2,010 genes differentially expressed between treated samples (n = 11) and control samples (n = 11)] (24). **(G)** Heatmap of average gene expression levels of *TREM1* and select target genes in myeloid, cancer, and fibroblast cell populations of individual tumors. Tumor samples (columns) are ranked left to right (descending order) according to the percentage of *TREM1*-expressing cells in the myeloid compartment.

To more precisely characterize *TREM1* expression in the breast tumor microenvironment, we profiled 770 cells from a fresh surgical TNBC specimen by single-cell (sc) RNAseq. Of 8 cell types identified by clustering (**Figures 6B, C** **)**, prevalent and high-level *TREM1* expression was observed in the myeloid cell population (n=301 cells), while low-level *TREM1* was observed sparsely in tumor cells and T cells (**Figure 6D**, left panel). Notably, *TREM1* expression overlapped sharply with markers of M2 macrophages (*MRC1/CD206*) and polymorphonuclear (PMN)-MDSCs (*OLR1*) (**Figure 6D**). To substantiate these findings, we analyzed a large publicly available scRNAseq dataset that profiled 175,942 cells from 31 breast tumors biopsied prior to treatment (38). Consistent with the initial scRNAseq and TREM-1 IF staining analyses, the prevalence and level of *TREM1* expression in the Bassez et al. dataset was greatest in the myeloid compartment, with sparse expression observed in other cell types, including cancer cells and fibroblasts, and to a lesser extent, B cells and endothelial cells (**Figures 7A, B** **)**. Similar *TREM1* expression patterns were observed among cell types grouped by tumor subtype (TNBC, n=13; ER+, n=15; HER2+ (ER+/-), n=3) (**Supplemental Figure 4**), and within tumors of individual patients (**Supplemental Figure 5**), confirming that *TREM1* expression in breast cancer is a predominantly myeloid phenomenon. Within the myeloid compartment (n=16,485 cells), *TREM1* was significantly overexpressed in cells positive for expression of *OLR1* or *MRC1* expression, as compared to cells negative for marker expression, respectively (**Figure 7C**; *P*<0.0001, both comparisons), consistent with our initial observations in the TNBC specimen (**Figure 6**), as well as the positive correlations observed between *TREM1* and *OLR1* and *MRC1* in the TCGA cohort (**Figure 5**).

To gain insight into the transcriptional programming downstream of TREM-1, we performed differential expression analysis in the myeloid cell population differentiated by *TREM1* expression (positive *vs*. negative). First, we observed a number of significantly differentially expressed genes that were reproducibly identified between the TNBC specimen dataset and that of Bassez et al. (**Figures 7D, E** **)**. Cross-referencing these genes with genes previously identified by Dower and colleagues (24) as being significantly induced by TREM-1-specific activation in human monocytes isolated from peripheral blood (**Figure 7F**), resulted in a list of confirmed transcriptional targets robustly overexpressed in the *TREM1+* cells of the breast cancer TME. Among these genes were known targets of TREM-1 signaling with pro-inflammatory functions (*IL1B*, *CCL7*, *CXCL3*) and known targets with anti-inflammatory or mixed pro- and anti-inflammatory functions (*IL1RN*, *INHBA*, *IL6*, *IL8*, *CCL2*). Also identified in the *TREM1+* positive myeloid cells of the breast tumors were genes shown by Dower et al. to be uniquely upregulated in monocytes by TREM-1-specific activation (by agonist antibody ligation) but not by LPS stimulation, including *MMP19*, *IL1RN*, *PLAUR*, *CCL7* and *SPP1*. Notably, *SLC11A1* and *SPP1* were among the top-most differentially expressed genes in all three datasets. Finally, in tumor myeloid cells negative for *TREM1* expression, we observed consistently higher expression of MHC class II antigen-presenting genes (*HLA-DRA*, *HLA-DRB1*, *HLA-DMB*, *HLA-DPA1*, *HLA-DQA1*) and the Th1-associated chemokine, *CXCL9*. However, these genes were not observed to be down-regulated by TREM-1 activation in the Dower et al. study, suggesting they represent a phenotype of non-TREM1 expressing cells in the breast TME. Of note, genes encoding canonical immunosuppressive factors such as TGF-β (*TGFB1*), IL-10 (*IL10*) and PD-L1 (*CD274*) were not consistently differentially expressed in our analyses; although IL10 and CD274 were moderately associated with TREM1 expression (**Figure 7D**) or activation (**Figure 7F**), respectively, these observations lacked reproducibility across datasets.

Next, we assembled a TREM-1 target gene panel comprising genes significantly induced by TREM-1 activation (in **Figure 7F**) and associated with intratumoral *TREM1+* myeloid cells (in **Figure 7E**) to serve as an estimator of TREM-1 signaling output in different cell and tumor populations.

Where *TREM1* expression was significantly higher in *OLR1+* or *MRC1+* cells as compared to cells negative for *OLR1* or *MRC1* expression (**Figure 7C**), the TREM-1 target genes also exhibited a simultaneous increase in frequency and magnitude of expression in the *OLR1+* or *MRC1+* cell compartments (**Supplemental Figure 6**), consistent with heightened TREM-1 signaling in these populations. We next used the target gene panel to profile individual tumors and major cell types expressing *TREM1*, including myeloid cells, cancer cells and fibroblasts (**Figure 7G**). By ranking tumor samples (columns) on *TREM1* expression frequency in the myeloid compartment, a clear correlation was observed between the percentage of *TREM1*-expressing myeloid cells and the frequency and magnitude of expression of the target genes. Moreover, we observed that myeloid *TREM1* frequency also associated with clinical breast cancer subtype (TNBC, ER+, HER2+), whereby an overall difference in rank of the percentage of *TREM1*-expressing cells was significant (*P*=0.03, three-group Kruskal-Wallis test). This difference in rank was also significant for the TNBC and ER+ subtypes alone (*P*=0.007, two-group comparison), where higher *TREM1* frequency associated with TNBC status while lower *TREM1* frequency associated with ER-positive tumor status. Notably, this observation was consistent with our findings in the MC1 and MC2 cohorts where *TREM1* expression by bulk tumor analysis was higher in basal-like tumors as compared to luminal tumors (**Figures 2C–F** **)**. Across individual tumors, we examined the frequencies and expression levels of *TREM1* and its target genes in cancer cells and fibroblasts (**Figure 7G**) where sparse, yet moderate to high *TREM1* expression could be detected in some cells (**Figure 7B**). In contrast to myeloid cells, neither the cancer cell nor fibroblast compartments showed high frequency *TREM1* expression in individual tumors or coordinated expression of target genes. Similar results were observed for the B cell and endothelial compartments (data not shown). Together, these findings suggest that TREM-1 signaling is frequently operative in breast cancer, and that myeloid cells are the predominant source of TREM-1 signaling in the TME.

Finally, we investigated the relationships between *TREM1* myeloid expression and the expression of other myeloid receptors related to TREM-1 activation and function. Historically, TREM-1 signaling has been widely studied in the context of lipopolysaccharide (LPS)-induced Toll-like receptor (TLR) signaling in innate immunity, where TLR activation *via* PAMP/DAMP-sensing can potently induce the expression and activation of TREM-1 in a pro-inflammatory amplification loop (21). In the Bassez et al. breast tumor myeloid cells, the expression of 5 TLR genes (*TLR1*, *TLR2*, *TLR4*, *TLR7* and *TLR8*) were detected in >10% of cells and at moderate to high expression levels. Upon comparing *TREM1* expression levels between myeloid cells positive or negative for TLR expression (**Supplemental Figure 7A**), no TLRs exhibited a substantial positive association with *TREM1* expression, suggesting that TLRs are not major determinants of *TREM1* expression in the breast TME. The TREM-2 receptor is a TREM family member with strong homology to TREM-1, known for its various roles in anti-inflammatory signaling (50) which may antagonize TREM-1 activity (51). Like TREM-1, TREM-2 also requires binding to the DAP12 adaptor protein to mediate signal transduction (52), thus potentially competing with TREM-1 in the presence of activating signals. *TREM2* expression was observed in approximately one-third of breast tumor myeloid cells, but with no significant association with *TREM1* expression (**Supplemental Figure 7B**). Moreover, in myeloid cells either positive or negative for *TREM2*, *TREM1+* cells were consistently associated with greater frequency and magnitude of expression of the TREM-1 target genes (**Supplemental Figure 7C**) consistent with TREM-1 activation occurring independent of TREM-2 status. Together, these findings suggest that neither TLRs nor TREM-2 substantially influence *TREM1* expression characteristics in the breast TME.

## Discussion

During acute inflammation, myeloid TREM-1 signaling stimulates production of cytokines and other factors that drive the expansion and survival of myeloid and lymphoid cells involved in pathogen clearance (21, 22, 53–55). This potent, but typically short-lived inflammatory response has been associated with M1 polarization and initiation of Th1 immunity (56, 57). However, in chronic infection and cancer, persistent activation signals and unresolved inflammation drive the production and accumulation of MDSCs with immunosuppressive properties (58, 59). Under such conditions, TREM-1 function is poorly understood.

Historically, TREM-1 signaling has been widely studied in the context of lipopolysaccharide (LPS)-induced TLR signaling, where TLR activation leads to dual TLR/TREM-1 signaling and subsequent M1-like polarization (24). Dower and colleagues investigated the cellular consequences of TREM-1 activation in human monocytes (24). They compared gene expression profiles of TREM-1-specific activation, where TREM-1 was activated by ligation with a TREM-1-specific *agonist* antibody (*αTREM-1*), to that of LPS-induced TLR/TREM-1 signaling, or the combination of the two. While the treatments similarly induced expression of certain TREM-1 cytokines, the authors noted that where LPS/TLR signaling showed bias toward expression of M1 markers, TREM-1-specific activation (without LPS) induced expression of TNFSF14 (M2 marker) and PPARG (required for M2 maturation) consistent with an alternative form of activation. Further, the authors noted that TREM-1 hyperactivation strongly repressed expression and secretion of IL-12 which was otherwise strongly induced by LPS/TLR activation. IL-12 is an M1 cytokine and potent promoter of anti-tumor immunity (60). Thus, the authors concluded that while LPS-induced TLR/TREM-1 signaling promotes the classical M1 phenotype, TREM-1-specific activation imparts a skewed phenotype with immunoregulatory properties. TREM-1-specific activation is believed to occur through direct binding of TREM-1 with its known ligands, HMGB1 and PGLYRP1 (43). Notably, many cancers types are known to express HMGB1 (61), including breast cancers (62, 63), suggesting TREM-1 hyperactivation could be a common feature of the tumor microenvironment.

To date, only a handful of studies have investigated roles for TREM-1 in cancer. In non-small cell lung cancer (NSCLC) and hepatocellular carcinoma (HCC), TREM-1 protein levels significantly correlate with poorer overall and disease-free survival (64, 65). In NSCLC, TREM-1 expression in tumors was restricted to tumor-infiltrating CD68+ myeloid cells (64), yet soluble TREM-1 in serum was also reported to have prognostic significance (66). In glioblastoma, a hypoxia-inducible inflammatory gene signature inclusive of myeloid-derived *TREM1* and *IL8* expression was significantly associated with poorer overall survival of patients (67). Similarly, in colorectal cancer (CRC), a risk score based on 19 genes transcriptionally regulated by TREM-1 and/or CTGF activation was found to be an independent risk factor for cancer recurrence and a significant prognostic indicator that may associate with reduced response to adjuvant chemotherapy (68).

In this report, we present first evidence that high *TREM1* expression in breast tumors is associated with inferior clinical outcomes of patients. *TREM1* expression in breast tumors was predominantly associated with the tumor-infiltrating myeloid compartment, and while *TREM1* was observed in all breast cancer subtypes, it was expressed to a higher degree in the non-luminal Basal-like and HER2E subtypes, suggesting that yet unknown molecular properties of these tumors may be more conducive to TREM-1 expression and downstream activation. Higher *TREM1* expression was robustly associated with worse distant metastasis-free survival in both ER-negative (basal-like) and ER-positive (Luminal A/B) breast cancers. In the presence of conventional prognostic markers, *TREM1* expression remained significantly associated with DMFS, indicating that *TREM1* contributes additive prognostic information not captured by conventional variables.

We also observed evidence that *TREM1*’s inferior outcome association in breast cancer may depend, in part, on an otherwise favorable immunological context. Previously described FID tumors are defined by the heightened expression of immune genes that reflect abundant effector cell infiltrates and equate with significantly better chemotherapy response and recurrence-free survival as compared to WID and PID tumors (8, 14, 20). Here, we found that the therapy-predictive power of *TREM1* was most prominent in the FID subclass, as evidenced by the significant relationship between low *TREM1* expression and a 1.9-fold increase in the relative frequency of response to neoadjuvant chemotherapy as compared to FID tumors with high *TREM1* expression. Similarly, we observed that high *TREM1* expression associated with inferior DMFS, and that this association was most prominent in breast tumors that otherwise exhibited a survival advantage in the context of high CD8+ T cell infiltration (as estimated by CIBERSORT). These observations are consistent with a model where *TREM1* expression reflects a pro-tumorigenic biology that, in part, antagonizes immune effector cell-mediated cancer control.

TREM-1 is known to signal through its signaling partner DAP12, which transactivates expression of numerous cytokines that chiefly include IL-1β, IL-6, IL-8, MCP-1/CCL2, M-CSF, and TNF-α (21). These cytokines comprise the inflammatory effectors of TREM-1 responsible for the pathological effects of TREM-1 signaling described in septic shock, rheumatoid arthritis, atherosclerosis, myocardial infarction, inflammatory bowel disease and retinal neovascular disorders (43, 69). Strikingly, these same cytokines overlap extensively with those required for the function and trafficking of MDSCs in cancer. In breast and other cancer types, MDSCs promote cancer cell growth, suppress anti-tumor immunity and antagonize immunotherapy responsiveness (70–72). MDSCs are recruited to tumors by the TREM-1 related cytokines IL-8, IL-6 and MCP-1/CCL2 (73–75). In the TME, IL-1β, IL-6, TNF-α and M-CSF are known to promote intratumoral MDSC expansion as well as activation of MDSC immunosuppressive functions (76–83). Moreover, the targeted inactivation of IL-6, MCP-1/CCL2 or M-CSF has been shown to augment anti-tumor immunity and reverse resistance to immunotherapy (84–88). More recently, TREM-1 signaling was reported to directly promote immunosuppression in hepatocellular carcinoma and abrogate immune checkpoint inhibition. In this study, TREM-1 signaling in TAMs was shown to impair anti-tumor activity of CD8+ T cells through the recruitment and activation of T-regs, thereby inducing resistance to anti-PD-L1 treatment (44). Together, these observations suggest that chemotactic signals downstream of TREM-1 activation could functionally contribute to immunosuppressed states in the TME.

In our analysis of the TCGA breast tumor cohort, we observed significant positive correlations between *TREM1* expression and the expression of TREM-1 target cytokines (*IL1B*, *IL8*, *IL6* and *CCL2*) and markers of MDSCs and TAMs (*CD11B/ITGAM*, *OLR1*, *CD14* and *CD206/MRC1*). By analyzing single-cell sequencing datasets, we further characterized cellular aspects of *TREM1* expression in breast tumors and identified *TREM1* transcriptional correlates indicative of myeloid TREM-1 activation in the breast TME and candidate mechanisms of pro-tumorigenic growth. In the breast TME, *TREM1* expression was observed predominantly in myeloid cells that expressed markers of MDSCs and TAMs. While *TREM1* expression was also observed in other cellular compartments, such as cancer cells and fibroblasts (as corroborated by IF staining results), *TREM1* expression in non-myeloid cell types was sparse, and showed no correlation with downstream TREM-1 targets. In both intra-tumoral myeloid cells and monocytes induced for TREM-1 activation by Dower et al. (24), *TREM1* was robustly associated with increased expression of known TREM-1 target genes (*IL1B*, *CCL7*, *CXCL3*, *IL1RN*, *INHBA*, *IL6*, *IL8*, *CCL2*), including genes with anti-inflammatory functions not generally associated with the conventional view of TREM-1 pro-inflammatory signaling in innate immunity. For example, *IL1RN* encodes the Interleukin 1 Receptor Antagonist cytokine that competes with IL-1 cytokines (IL-1α and IL-1β) to inhibit IL-1 signaling. As IL-1 signaling can enhance Th1 polarization, promote activation of CD8+ T cells, and facilitate T cell priming by dendritic cells (89), *IL1RN* could play an immune suppressive role in the TME. *INHBA* encodes the TGF-β family member Activin A, which has been reported to: 1) impair anti-tumor immunity by inhibiting NK cell proliferation and granzyme B production (90); 2) promote regulatory T cell infiltration into tumors treated with radiotherapy and TGF-β blockade (91); and 3) function as a Th2 cytokine to induce macrophage M2 polarization (92). *SPP1*, which encodes the cytokine Osteopontin, was identified in our study as one of only several genes ranked in all three datasets as a top-most significantly differentially expressed gene. Interestingly, in the Dower et al. study, *SPP1* expression and Osteopontin protein levels were shown to be highly induced by TREM-1-specific activation, but not by LPS/TLR stimulation. Osteopontin, through interaction with the CD44 receptor, modulates inflammatory signaling in T cells and macrophages, and can induce migration, proliferation and survival of non-lymphoid cells. While Osteopontin can enhance activation and survival of T cells in graft-versus-host disease (93), in cancer models, Osteopontin is a newly identified immune checkpoint expressed by intra-tumoral myeloid cells that upregulates PD-L1 expression (94) and directly inhibits activation and proliferation of tumor-reactive CD8+ T cells, thereby blocking anti-tumor immunity (95). Thus, it is feasible that TREM-1 signaling in the breast TME could, through multiple different mechanisms, contribute to the blockade of specific anti-tumor immune responses. Importantly, key anti-inflammatory molecules characteristic of TAMs and MDSCs, such as TGF-β (*TGFB1*), IL-10 (*IL10*) and PD-L1 (*CD274*), were not consistently associated with *TREM1* expression in tumors and TREM-1 activation in monocytes. These genes were also not identified among the negative immune interactors associated with reduced neoadjuvant chemotherapy response in the MDACC-701 dataset, nor were they identified with the inferior DMFS-associated genes correlated with *TREM1* expression in the MC1 cohort. A plausible explanation is that these anti-inflammatory factors are not direct targets of TREM-1, or are not solely modulated by TREM-1, and therefore, are likely not major contributors to TREM-1-related tumor phenotypes.

TREM-1 paracrine signaling may also activate tumor-intrinsic pathways. MDSC-secreted factors that overlap with TREM-1 target genes, including IL-1β, TNF-α, IL-8 and IL-6, have been reported to promote angiogenesis and induce cancer cell migration, invasion, and survival (96). In co-culture experiments, TREM-1 signaling was reported to induce migration of liver cancer cells, which could be abrogated by incubation with a TREM-1-blocking peptide (65). In T-cell-deficient, but myeloid-competent mouse xenograft models, TREM-1 blockade inhibited infiltration of TAMs into pancreatic tumors, and significantly reduced formation of both human pancreatic and lung tumors (97, 98). A role for TREM-1 signaling in tumor formation is supported by studies in *TREM1* knockout mice that model inflammation-driven liver cancer (99) and CRC (100). In the latter studies, TREM-1 deficiency delayed tumor formation with concomitant decreases in IL-1β and IL-6 (100). TREM-1 deficiency also reduced the number of tumor-infiltrating neutrophils in both models, with alterations observed in cytokine and chemokine profiles (100). These reports, together with our findings in breast cancer, support the emerging view that TREM-1 signaling may contribute to immunosuppressive and tumor-intrinsic cancer-promoting pathways, and may represent an oncogenic axis common to many cancer types.

Several limitations of this study are noteworthy. First, our interpretations regarding the role of TREM-1 in breast cancer are based solely on correlative analyses, statistical associations and inferences drawn from other cancer research. Second, from our studies, we cannot rule out the possibility that TREM-1 activation may, in certain instances, promote anti-tumor immunity. For example, it is possible that in some tumors, TREM-1 signaling could be triggered downstream of TLR activation which may induce a more M1-like polarization in monocytes (24) and therefore contribute to a pro-inflammatory shift in the TME; however, our findings of a lack of robust association between myeloid *TREM1* and *TLR* gene expression suggests that such a phenomenon is not common in breast tumors. Third, how other pathological processes and pathways operative in breast cancer correlate with TREM-1 expression, the immune subclasses, or clinical endpoints cannot be comprehensively determined from this study. TREM-1 functional studies that take advantage of TREM-1-activating antibodies (101, 102) and inhibitory peptides (44, 52) in the context of murine immuno-oncology models will be necessary to test the hypotheses presented here, and to establish the therapeutic value of targeting TREM-1 in the clinical management of breast cancer.

## Conclusions

This work demonstrates, for the first time, that *TREM1* expression in breast tumors associates with a reduced interval to clinical metastasis and a decreased responsiveness to neoadjuvant chemotherapy. While *TREM1* expression positively correlates with markers of alternatively activated macrophages and MDSCs, as well as known cytokine targets downstream of TREM-1 activation, *TREM1* negatively correlates with markers of anti-tumor immunity and exhibits associations with inferior clinical outcomes in tumors with otherwise advantageous immune characteristics. Our findings fit a model where myeloid-derived TREM-1 signaling is operative in human breast tumors and antagonizes anti-tumor immune processes. Further study of the role of TREM-1 inhibition in breast cancer as a tool to overcome resistance to immune checkpoint blockade is warranted.

## Data Availability Statement

Raw and processed data files have been deposited in the Gene Expression Omnibus (GEO; www.ncbi.nlm.nih.gov/geo/) and can be accessed through GEO Series accession number GSE188600.

## Ethics Statement

The studies involving human participants were reviewed and approved by Wake Forest University Health Sciences Institutional Review Board. The patients/participants provided their written informed consent to participate in this study.

## Author Contributions

AP and LM conceived of the study and analytical strategies. Data analyses were performed by AP, KZ, JC, JS, GJ, JWC, QS, MB, and ER, AP, MS, MH-M and QS conducted the scRNA-seq studies. AP and LM wrote the paper with critical input and technical guidance from CL, KZ, AS, YL, AT, DB, CP, and MB. All authors critically reviewed the findings, edited the paper and approved the final version to be published.

## Funding

This work was supported by the American Cancer Society (RSG-12-198-01-TBG; to LM), the Mary Kirkpatrick Professorship for Breast Cancer Research (to LM) and the NIH (T32-CA079448 Cancer Biology Training Grant; to JC). This study was also supported by the Wake Forest Baptist Compressive Cancer Center’s Cancer Genomics Shared Resource (CGSR), Bioinformatics Shared Resource (BISR), and Tumor Tissue and Pathology Shared Resource (TTPSR) funded by the National Cancer Institute’s Cancer Center Support Grant award number P30CA012197. This work was also partially supported by the Indiana University Precision Health Initiative (to JS).

## Author Disclaimer

The content of this publication is solely the responsibility of the authors and does not necessarily represent the official views of the National Cancer Institute.

## Conflict of Interest

AS is an employee of SignaBlok, Inc. LM has an advisory role for Bristol Myers Squibb. AT has research funding (to the institution) from Sanofi, stock ownership of Gilead Sciences, Johnson and Johnson, Bristol Myers Squibb and Pfizer, and advisory roles for BeyondSpring and Lilly.

The remaining authors declare that the research was conducted in the absence of any commercial or financial relationships that could be construed as a potential conflict of interest.

## Publisher’s Note

All claims expressed in this article are solely those of the authors and do not necessarily represent those of their affiliated organizations, or those of the publisher, the editors and the reviewers. Any product that may be evaluated in this article, or claim that may be made by its manufacturer, is not guaranteed or endorsed by the publisher.
