# Supplementary material for: Bulk and Single-Cell Profiling of Breast Tumors Identifies TREM-1 as a Dominant Immune Suppressive Marker Associated With Poor Outcomes
Source: Front Oncol. 2021 Dec 8;11:734959. doi: 10.3389/fonc.2021.734959 (PMC8692779; doi:10.3389/fonc.2021.734959)
Supplement: Supplementary file 1 [file DataSheet_1.pdf]

![frontiers]

Supplementary Material

Supplementary Figures 1 – 7

Supplementary Table 1

Supplementary Figure 1

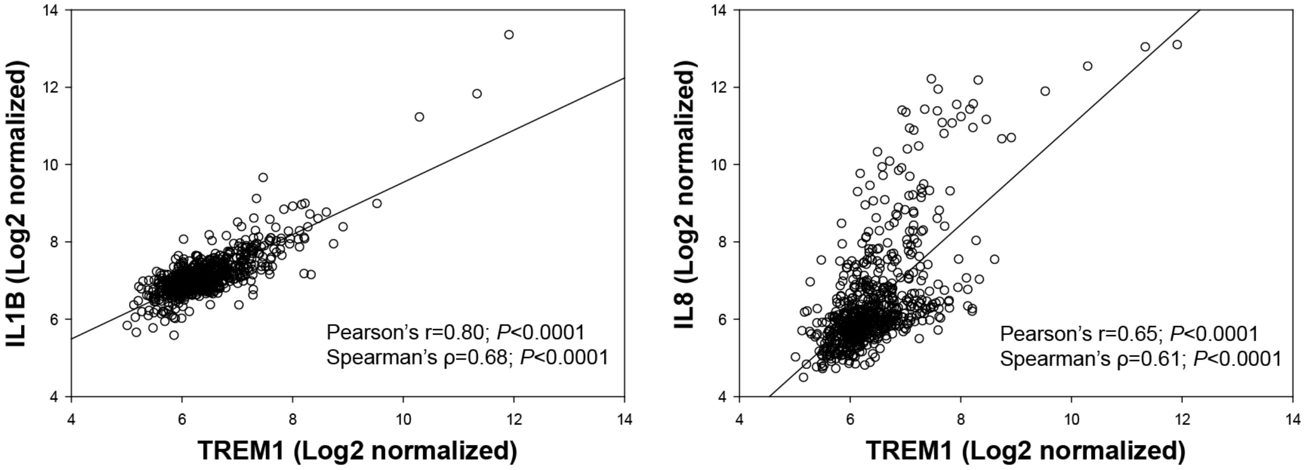

Supplemental Figure 1. *TREM1* expression correlates significantly with expression of *IL1B* (left panel) and *IL8/CXCL8* (right panel) in the MDACC-701 dataset. Pearson's and Spearman correlation coefficients and p-values are shown.

Supplementary Figure 2

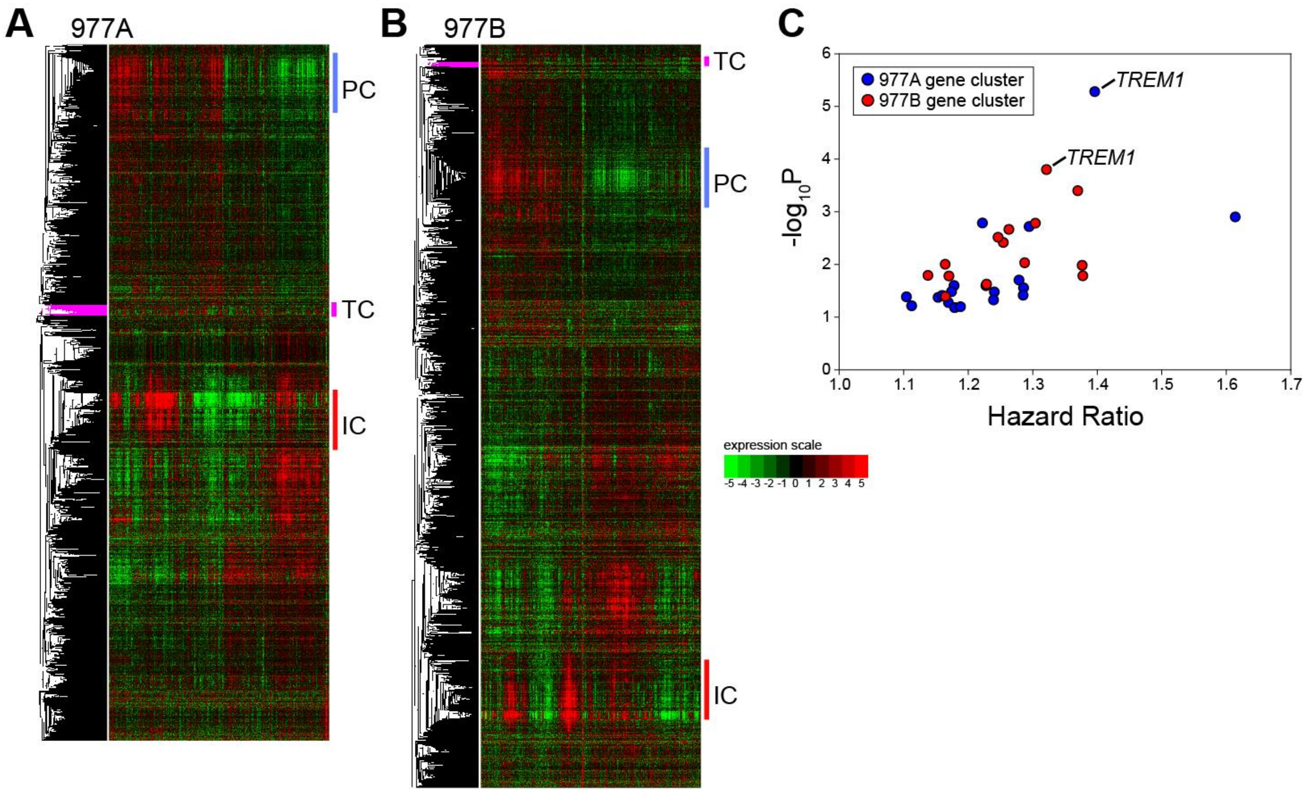

Supplemental Figure 2. Discovery of a *TREM1*-associated gene cluster associated with poor DMFS. Breast tumor expression profiles of the MC1 meta-cohort were randomized to two subgroups, comprising 977 tumors in each, for testing and validation purposes (977A and 977B, respectively). Within each subgroup, genes with significant associations with DMFS by Cox regression (q<0.1, Benjamini-Hochberg) were selected for hierarchical clustering (977A, n = 3,094 genes; 977B, n = 3,304 genes). Genes corresponding to the proliferation gene cluster (PC, blue bar), immune gene clusters (IC, red bar) and the *TREM1*-associated gene cluster (TC, pink bar) are shown to the right of the heat maps for **(A)** 977A and **(B)** 977B. Tumors are represented by columns (dendrograms omitted); genes are represented by rows. Red indicates above-mean expression; green indicates below-mean expression. **(C)** Genes within the *TREM1*-associated gene clusters having average Pearson correlation of 0.28 in 977A (blue circles, n = 19 genes) and 977B (red circles, n = 14 genes) were compared by plotting Cox regression hazard ratios (X-axis) and false discovery rate-adjusted, negative $\log_{10}$ p-values (Y-axis).

Supplementary Figure 3

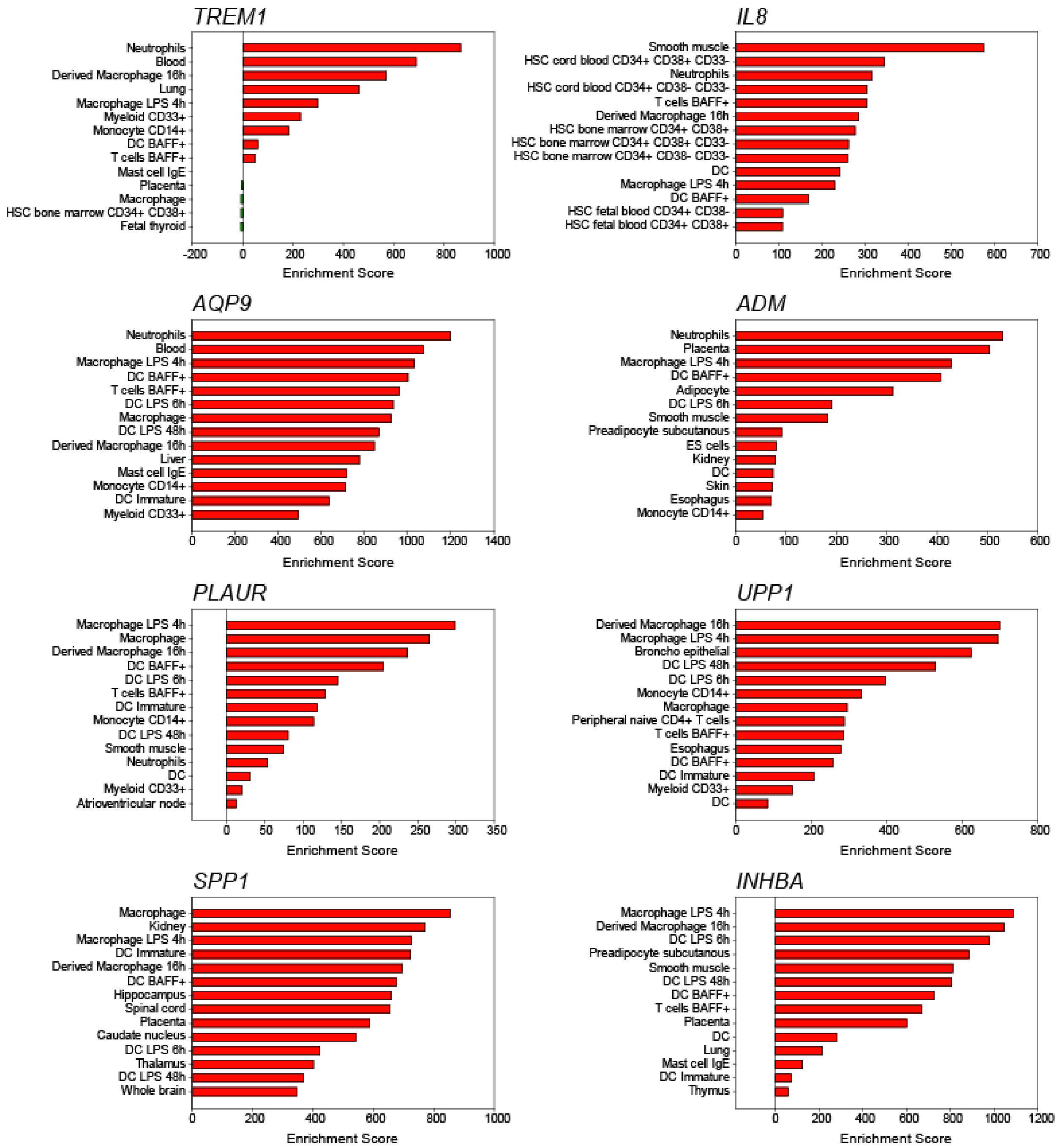

Supplemental Figure 3. *TREM1* and co-expressed genes in breast tumors exhibit enriched expression in cells of the myeloid lineage. Tissue-specific enrichment scores were generated according to methods described by Benita et. al. (Benita et al., 2010) for >14,000 genes comprising the Affymetrix U133A GeneChip and 126 human cell subsets and tissues. For each gene, the top 14 most enriched cell subsets and tissues are shown.

Supplementary Figure 4

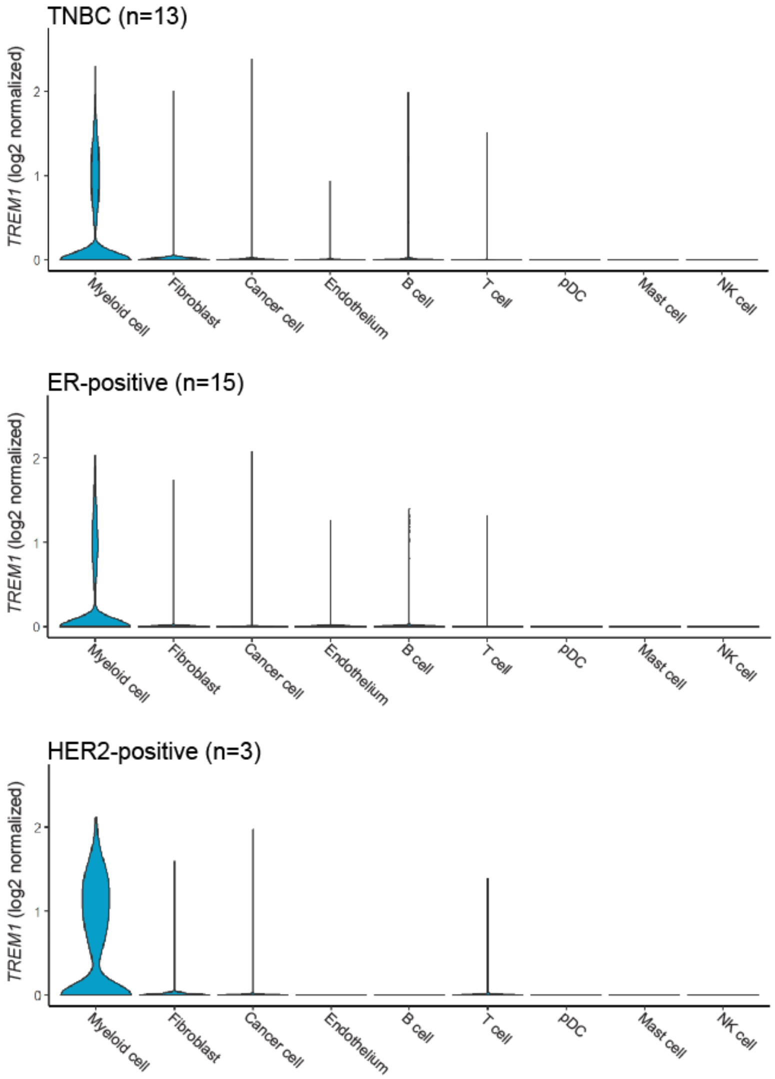

Supplemental Figure 4. *TREM1* expression in cellular subsets of the Bassez et. al. scRNAseq dataset grouped by breast cancer subtype.

Supplementary Figure 5

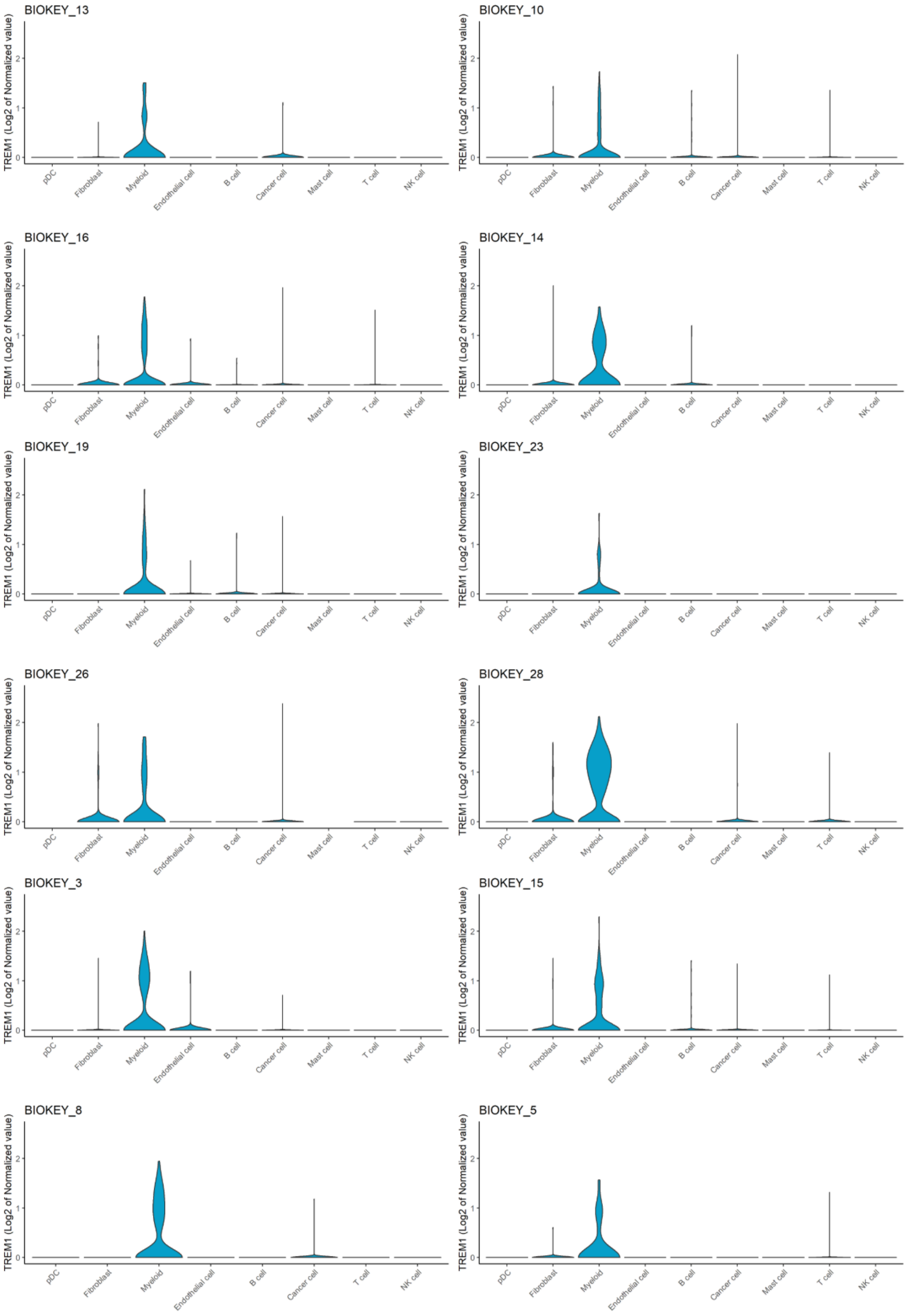

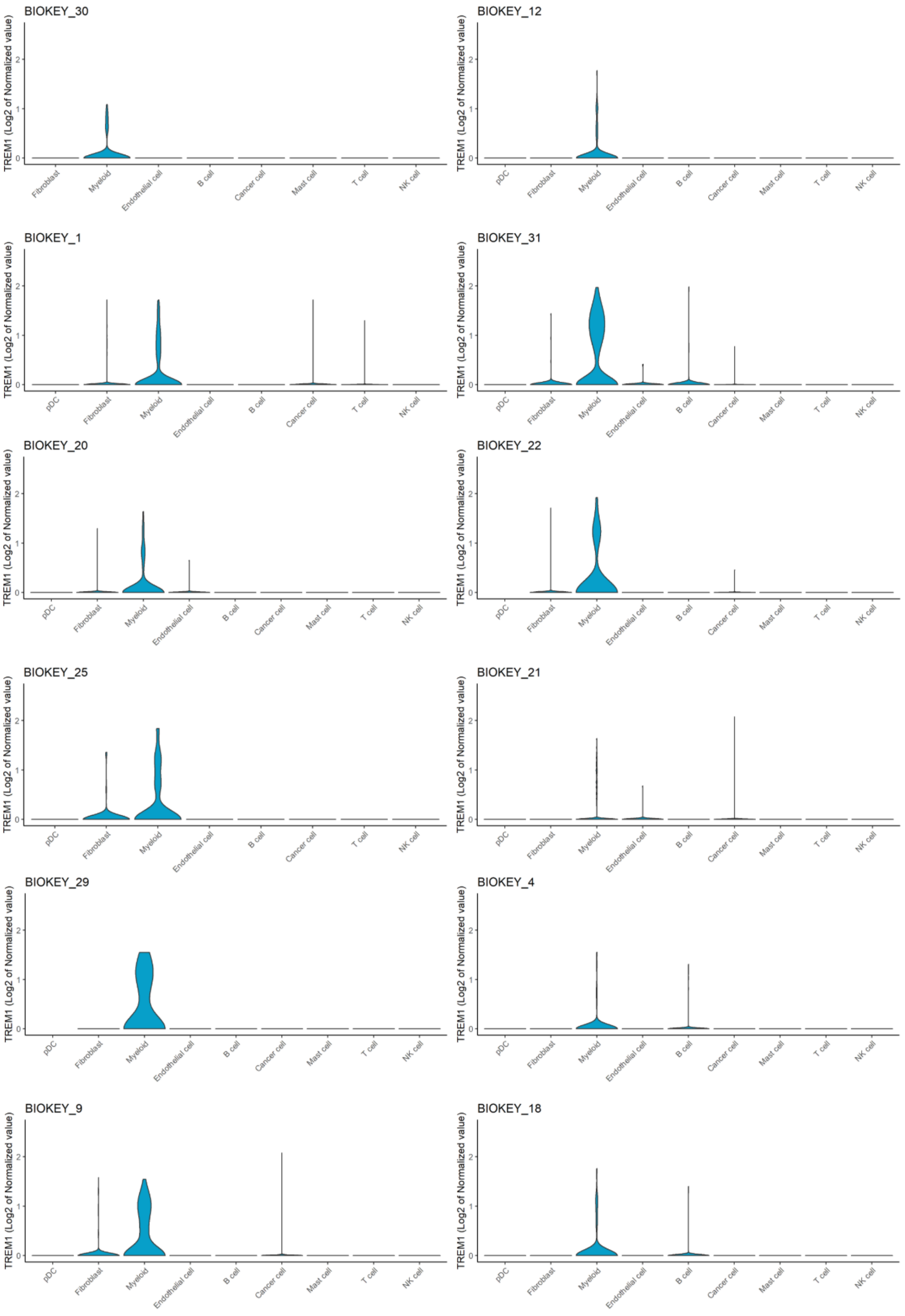
BIOKEY_30
BIOKEY_12
BIOKEY_1
BIOKEY_31
BIOKEY_20
BIOKEY_22
BIOKEY_25
BIOKEY_21
BIOKEY_29
BIOKEY_4
BIOKEY_9
BIOKEY_18
TREM1 (Log2 of Normalized value)
0
1
2
pDC
Fibroblast
Myeloid
Endothelial cell
B cell
Cancer cell
Mast cell
T cell
NK cell

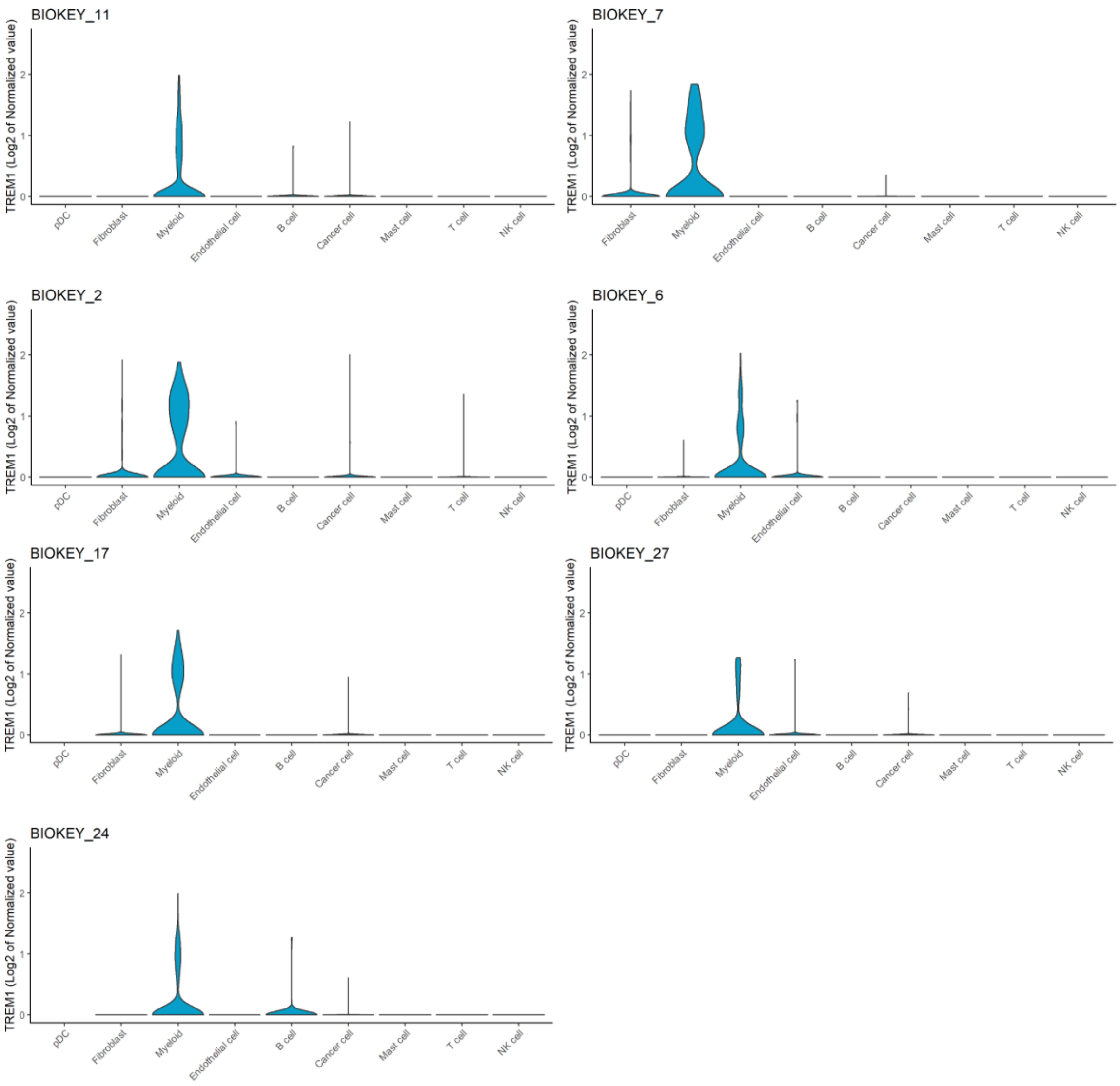

Supplemental Figure 5. ***TREM1*** **expression in cellular subsets of the Bassez et. al. dataset grouped by individual breast cancer patients (n=31).**

Supplementary Figure 6

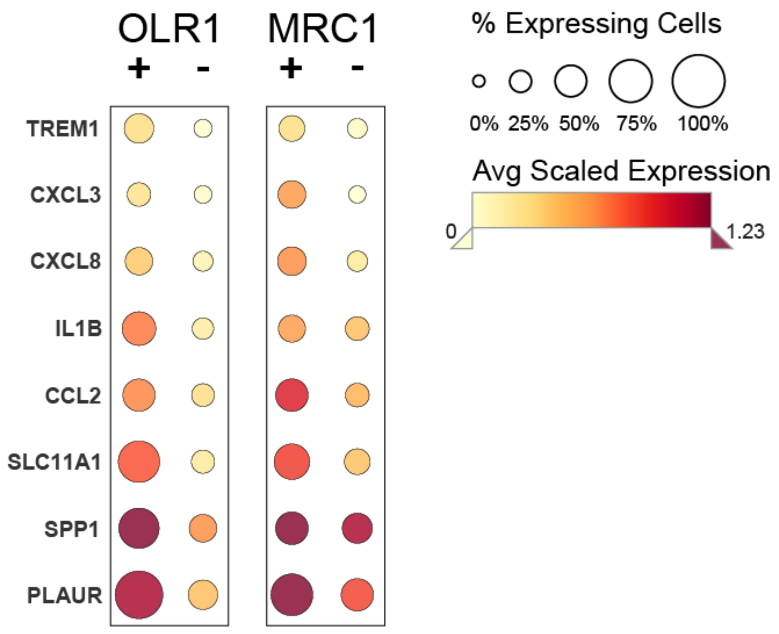

Supplemental Figure 6. *TREM1* target gene expression levels are enriched in *OLR1*+ or *MRC1*+ myeloid cells relative to myeloid cells negative for marker gene expression. *TREM1* target gene frequencies of expression (% expressing cells) and average expression levels (heatmap) are shown compared between marker gene positive and negative myeloid cells of the Bassez et. al. dataset.

Supplementary Figure 7

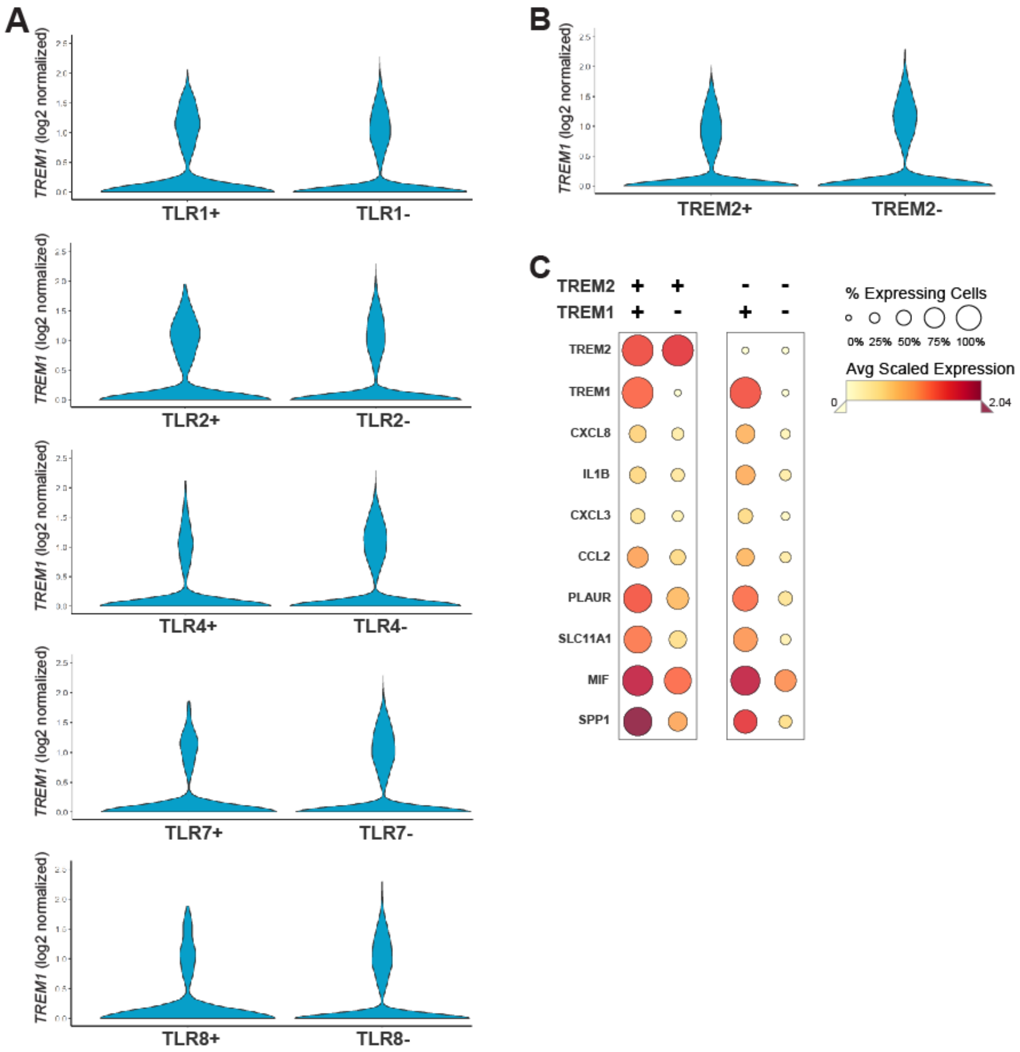

Supplemental Figure 7. Analysis of *TREM1* myeloid expression and expression of myeloid receptors related to TREM-1 activation and function. In the myeloid compartment of the Bassez et. al. dataset, **(A)** *TREM1* expression distributions are shown compared between *TLR+* and *TLR–* myeloid cell populations, and **(B)** *TREM2+* and *TREM2–* myeloid cells. **(C)** *TREM1* target gene frequencies of expression (% expressing cells) and average expression levels (heatmap) are shown compared between *TREM2+* and *TREM2–* myeloid cells.

Supplemental Table 1. Genes and corresponding Z values identified by logistic regression as having statistical negative interactions with immune-associated neoadjuvant chemo response in the MDACC-701 dataset.

Probest ID	Gene Symbol	Binary_Z	Neutrophil Chemotaxis
202155_s_at	NUP214	-4.1	
217336_at	RPS10	-3.9	
218955_at	BRF2	-3.4	
44822_s_at	MIER2	-3.3	
37254_at	ZNF133	-3.3	
219434_at	TREM1	-3.3	X
207205_at	CEACAM4	-3.1	
209495_at	CEP250	-3.1	
202573_at	CSNK1G2	-3.1	
204594_s_at	MIEF1	-3.1	
204828_at	RAD9A	-3.1	
206019_at	RBM19	-3.1	
216036_x_at	WDTC1	-3.1	
209797_at	CNPY2	-3.0	
32032_at	ESS2	-3.0	
221983_at	RETREG2	-3.0	
216505_x_at	RPS10P5	-3.0	
201320_at	SMARCC2	-3.0	
214783_s_at	ANXA11	-2.9	
205654_at	C4BPA	-2.9	
215568_x_at	[unknown]	-2.9	
203185_at	RASSF2	-2.9	
218141_at	UBE2O	-2.9	
220730_at	ZNF778	-2.9	
218274_s_at	ANKZF1	-2.8	
218457_s_at	DNMT3A	-2.8	
214185_at	KHDRBS1	-2.8	
212844_at	RRP1B	-2.8	
41657_at	STK11	-2.8	
217733_s_at	TMSB10	-2.8	
216409_at	ACSL6	-2.7	
218950_at	ARAP3	-2.7	
219261_at	C7orf26	-2.7	
221357_at	CHRM4	-2.7	
202500_at	DNAJB2	-2.7	
210736_x_at	DTNA	-2.7	
222113_s_at	EPS15L1	-2.7	
206885_x_at	GH1	-2.7	
210079_x_at	KCNAB1	-2.7	
221792_at	RAB6A	-2.7	
37462_i_at	SF3A2	-2.7	
221939_at	CARM1	-2.6	
207556_s_at	DGKZ	-2.6	
218660_at	DYSF	-2.6	
213242_x_at	KIAA0284	-2.6	
213281_at	LOC100288387	-2.6	
35147_at	MCF2L	-2.6	
215484_at	[unknown]	-2.6	
205656_at	PCDH17	-2.6	
219459_at	POLR3B	-2.6	
200869_at	RPL18A	-2.6	
207355_at	SLC1A7	-2.6	
214686_at	ZNF266	-2.6	
210062_s_at	ZNF589	-2.6	
214322_at	CAMK2G	-2.5	
220015_at	CASZ1	-2.5	
214627_at	EPX	-2.5	
210981_s_at	GRK6	-2.5	
216309_x_at	JRK	-2.5	
210058_at	MAPK13	-2.5	
202463_s_at	MBD3	-2.5	
221379_at	[unknown]	-2.5	
219039_at	SEMA4C	-2.5	
218596_at	TBC1D13	-2.5	
201768_s_at	CLINT1	-2.4	
220522_at	CRB1	-2.4	
214030_at	CRYBG3	-2.4	
219914_at	ECEL1	-2.4	
221539_at	EIF4EBP1	-2.4	
210043_at	FRMD8	-2.4	
214626_s_at	GANAB	-2.4	
220165_at	INO80D	-2.4	
49329_at	KLHL22	-2.4	
207908_at	KRT2	-2.4	
210252_s_at	MADD	-2.4	
207473_at	MLN	-2.4	
215200_x_at	[unknown]	-2.4	
202924_s_at	PLAGL2	-2.4	
220894_x_at	PRDM12	-2.4	
220758_s_at	ROBO4	-2.4	
209980_s_at	SHMT1	-2.4	
214096_s_at	SHMT2	-2.4	
209777_s_at	SLC19A1	-2.4	
211123_at	SLC5A5	-2.4	
216042_at	TNFRSF25	-2.4	
213822_s_at	UBE3B	-2.4	
37860_at	ZNF337	-2.4	
214760_at	ZNF337	-2.4	
216750_at	APBB2	-2.3	
214052_x_at	BAT2L2	-2.3	
214643_x_at	BIN1	-2.3	
219442_at	C16orf67	-2.3	
219161_s_at	CKLF	-2.3	
204971_at	CSTA	-2.3	
2028_s_at	E2F1	-2.3	
206429_at	F2RL1	-2.3	
60528_at	JMJD7	-2.3	
210635_s_at	KLHL20	-2.3	
37796_at	LRCH4	-2.3	
219320_at	MYO19	-2.3	
204448_s_at	PDCL	-2.3	
216458_at	PQLC3	-2.3	
219897_at	RNF122	-2.3	
212777_at	SOS1	-2.3	
207540_s_at	SYK	-2.3	X
78495_at	ZNF783	-2.3	
219623_at	ACTR5	-2.2	
219437_s_at	ANKRD11	-2.2	
219521_at	B3GAT1	-2.2	
210708_x_at	CASP10	-2.2	
206728_at	ECE2	-2.2	
205321_at	EIF2S3	-2.2	
204817_at	ESPL1	-2.2	
205650_s_at	FGA	-2.2	
220819_at	FRMD1	-2.2	
204973_at	GJB1	-2.2	
211977_at	GPR107	-2.2	
221297_at	GPRC5D	-2.2	
209558_s_at	HIP1R	-2.2	
219281_at	MSRA	-2.2	
217619_x_at	[unknown]	-2.2	
216551_x_at	PLCG1	-2.2	
201896_s_at	PSRC1	-2.2	
205228_at	RBMS2	-2.2	
211735_x_at	SFTPC	-2.2	
205592_at	SLC4A1	-2.2	
216271_x_at	SYDE1	-2.2	
213107_at	TNIK	-2.2	
218020_s_at	ZFAND3	-2.2	
205437_at	ZNF211	-2.2	
78330_at	ZNF335	-2.2	
220501_at	ACTL7A	-2.1	
90265_at	ADAP1	-2.1	
206105_at	AFF2	-2.1	
202206_at	ARL4C	-2.1	
212645_x_at	BRE	-2.1	
221764_at	C19orf22	-2.1	
206327_s_at	CDH15	-2.1	
219640_at	CLDN15	-2.1	
206970_at	CNTN2	-2.1	
208159_x_at	DDX11	-2.1	
208250_s_at	DMBT1	-2.1	
221812_at	FBXO42	-2.1	
208885_at	LCP1	-2.1	
205036_at	LSM6	-2.1	
43544_at	MED16	-2.1	
211599_x_at	MET	-2.1	
215292_s_at	MKL1	-2.1	
216554_s_at	[unknown]	-2.1	
217390_x_at	[unknown]	-2.1	
208360_s_at	[unknown]	-2.1	
209062_x_at	NCOA3	-2.1	
206402_s_at	NPFF	-2.1	
205261_at	PGC	-2.1	
204613_at	PLCG2	-2.1	
208286_x_at	POU5F1	-2.1	
204044_at	QPRT	-2.1	
216383_at	RPL18A	-2.1	
214370_at	S100A8	-2.1	X
203535_at	S100A9	-2.1	X
209381_x_at	SF3A2	-2.1	
215926_x_at	SNAPC4	-2.1	
52169_at	STRADA	-2.1	
203046_s_at	TIMELESS	-2.1	
215540_at	TRAC	-2.1	
211432_s_at	TYRO3	-2.1	
65521_at	UBE2D4	-2.1	
211682_x_at	UGT2B28	-2.1	
212156_at	VPS39	-2.1	
203892_at	WFDC2	-2.1	
207708_at	ALOXE3	-2.0	
219141_s_at	AMBRA1	-2.0	
221077_at	ARMC4	-2.0	
220659_s_at	C7orf43	-2.0	
207861_at	CCL22	-2.0	X
213523_at	CCNE1	-2.0	
216293_at	CLTA	-2.0	
214076_at	GFOD2	-2.0	
213180_s_at	GOSR2	-2.0	
204949_at	ICAM3	-2.0	
213450_s_at	ICOSLG	-2.0	
205067_at	IL1B	-2.0	X
202859_x_at	IL8	-2.0	X
211806_s_at	KCNJ15	-2.0	
207339_s_at	LTB	-2.0	
206571_s_at	MAP4K4	-2.0	
210059_s_at	MAPK13	-2.0	
202519_at	MLXIP	-2.0	
203036_s_at	MTSS1	-2.0	
210088_x_at	MYL4	-2.0	
213955_at	MYOZ3	-2.0	
216172_at	[unknown]	-2.0	
221382_at	[unknown]	-2.0	
222368_at	[unknown]	-2.0	
215279_at	[unknown]	-2.0	
217088_s_at	NCR1	-2.0	
221526_x_at	PARD3	-2.0	
215031_x_at	RNF126	-2.0	
201909_at	RPS4Y1	-2.0	
202917_s_at	S100A8	-2.0	X
221701_s_at	STRA6	-2.0	
216919_at	TP53I11	-2.0	
221906_at	TXNRD3	-2.0	
204598_at	UBOX5	-2.0	
210301_at	XDH	-2.0	
214713_at	YLPM1	-2.0	
214795_at	ZMYND8	-2.0	
